# Intraoperative somatosensory evoked potential (SEP) monitoring: an updated position statement by the American Society of Neurophysiological Monitoring

**DOI:** 10.1007/s10877-024-01201-x

**Published:** 2024-07-27

**Authors:** J. Richard Toleikis, Christopher Pace, Faisal R. Jahangiri, Laura B. Hemmer, Sandra C. Toleikis

**Affiliations:** 1 St. Charles, IL USA; 2 Limbic Neuro, LLC, New York, NY USA; 3 Global Innervation LLC, Dallas, TX USA; 4 Department of Neuroscience, School of Behavioral and Brain Sciences, University of Texas at Dallas, Richardson, TX USA; 5 Anesthesiology and Neurological Surgery, Northwestern University Feinberg School of Medicine, Chicago, IL USA

**Keywords:** Somatosensory evoked potentials, SSEP, SEP, Intraoperative neuromonitoring, IONM, Neurophysiological monitoring

## Abstract

Somatosensory evoked potentials (SEPs) are used to assess the functional status of somatosensory pathways during surgical procedures and can help protect patients’ neurological integrity intraoperatively. This is a position statement on intraoperative SEP monitoring from the American Society of Neurophysiological Monitoring (ASNM) and updates prior ASNM position statements on SEPs from the years 2005 and 2010. This position statement is endorsed by ASNM and serves as an educational service to the neurophysiological community on the recommended use of SEPs as a neurophysiological monitoring tool. It presents the rationale for SEP utilization and its clinical applications. It also covers the relevant anatomy, technical methodology for setup and signal acquisition, signal interpretation, anesthesia and physiological considerations, and documentation and credentialing requirements to optimize SEP monitoring to aid in protecting the nervous system during surgery.

## ASNM position statement endorsement

This document presents the updated American Society of Neurophysiological Monitoring (ASNM) position statement regarding the utilization of somatosensory evoked potentials (SEPs) for intraoperative monitoring purposes. This position statement is based on information published in the current scientific and clinical peer-reviewed literature and presented in previously published guidelines and position statements of various clinical societies. This document may not include all possible methodologies and interpretive criteria, nor is it intended to exclude any new innovations or developments that occur within the currently established protocols. Furthermore, ASNM recognizes this position statement as an educational service.

## Introduction

This position paper aims to address the relevant history, rationale, anatomy, methodology, anesthesia and physiologic considerations, applications, interpretation, documentation and credentialing associated with the use of SEPs for intraoperative neurophysiologic monitoring (IONM). SEPs have been used as an intraoperative monitoring tool for nearly 50 years or more [1–4]. Among evoked potentials, SEPs are the most utilized monitoring modality. They provide a means for functional assessment and localizing information about the somatosensory system. In addition, they act as a complement to the use of other IONM modalities, such as motor evoked potentials (MEPs).

### History

As early as the mid-1960s, Larson and Sances reported on the utilization of SEPs as a monitoring tool during neurosurgical procedures [1, 5]. Later, McCallum and Bennett [2], Nash et al. [3], and Tamaki and Kubota [4] reported on their utilization during spinal surgery. Their purpose was to act as a supplement to the use of the wake-up test, a procedure whose use was known to be associated with a number of possible hazards [6] and to provide a warning in the case of compromised spinal cord function. Soon after, their utilization was expanded to include various other surgical procedures when the brain, brainstem, or peripheral nerve function was placed at risk, such as for descending aortic procedures when there was a risk of spinal cord infarction [7, 8], and for vascular procedures such as carotid endarterectomy and intracranial aneurysm repair when there was a risk of cerebral infarction [9, 10]. A form of SEPs known as dermatomal SEPs or DSEPs was also introduced to assess nerve root function during surgery [11].

### Previous guidelines

Several guidelines and recommendations of various professional societies were developed and published for the intraoperative utilization and interpretation of SEPs. In 1987, the American Electroencephalographic Society (AEEGS), now the American Clinical Neurophysiology Society (ACNS), published the first of these guidelines [12]. In 1994, these were revised [13]. Other guidelines, recommended standards, and policy and position statements include those of the American Society of Electroneurodiagnostic Technologists (ASET) [14], the International Organization of Societies for Electrophysiological Technology (OSET) [15], the International Federation of Clinical Neurophysiology (IFCN) [16], the American Academy of Neurology (AAN), the American Clinical Neurophysiology Society (ACNS) [17–19], and the ASNM [20, 21]. More recently, additional recommendations for the intraoperative use of SEPs were published by the International Society of Intraoperative Neuromonitoring (ISIN) [22].

### Rationale and clinical basis for SEP monitoring

When used to assess function, SEP responses are typically elicited by stimulation of a mixed nerve at a peripheral site distal to the structure at risk. They may be recorded at both a distal location and one or more sites proximal to the structure at risk. The distal recording site is used to ensure adequate stimulation. The proximal recording sites are used to monitor changes that may occur with functional compromise of the structure in question.

The primary goal of SEP monitoring is to preserve neurological function. The intraoperative use of SEP monitoring helps to reduce the risk of injuring the dorsal column-medial lemniscus somatosensory system pathways associated with mediating proprioception, stereognosis, vibration sense, and discriminative touch (weight and two-point). SEPs are used to assess the functional status of somatosensory pathways during surgical procedures which may affect peripheral nerve or plexus [23–29], cauda equina and conus medularis tumor removal [30], spinal deformity correction, traumatic spinal fracture repair, tumor resection [4, 31–33], posterior fossa tumor removal [34], and brain function (carotid endarterectomy, aneurysm repair, tumors) [35, 36]. They are also used for mapping of the dorsal columns [37–43] or to identify the central sulcus by means of cortical mapping during removal of supratentorial brain tumors affecting eloquent areas [44–49]. In addition, SEPs can be used to optimize the placement of spinal cord stimulators [42, 43, 50].

SEP monitoring also provides a complement to the use of MEP monitoring for surgeries when corticospinal motor function is primarily at risk. When MEPs are not utilized, as was the case prior to their development and implementation, SEPs can still indirectly help to avoid motor injury because of the proximity of the motor and sensory pathways to each other. However, there are instances when a motor deficit may occur without any SEP deterioration and the opposite may also occur [50, 51].

## Anatomy and blood supply

### Anatomy

Application and interpretation of SEPs requires a detailed knowledge of the relevant anatomy and blood supply of the sensory pathway. The anatomy believed to mediate the short latency SEP pathway and its relevant blood supply, as synthesized, from multiple sources [52–59] is as follows. The SEP is primarily mediated by large diameter, myelinated, low-threshold, fast conducting axons that combine to form the distal sensory nerve and spinal cord tracts of the SEP pathway. They originate from sensory neurons whose cell bodies reside in the dorsal root ganglion (DRG), which are often defined as the first order neuron in the SEP pathway. Pseudounipolar neurons of the DRG are unique anatomically because their axon bifurcates such that a single process extends from the periphery to the central nervous system (CNS) passing the soma in the DRG along the way. The peripheral process of the DRG neurons innervates receptive sensory organs in the skin (such as Meisner’s corpuscles, Pacinian corpuscles, Merkel’s discs, Ruffini corpuscles, and free nerve endings), and in intrafusal and extrafusal spindle organs within muscles and tendons. The axon of the DRG neuron continues into the CNS as a central process before synapsing proximally. Action potentials, which originate at or near the distal sensory terminals, course along this same route passing the soma in the DRG. Axon collaterals from DRG neurons synapse with interneurons within the spinal cord gray matter participating in reflex arcs and modulating muscle tone.

Sensory fibers traverse toward the spinal cord by coursing through a plexus respective to their anatomic origin. In general, those from the distal lower limbs and genitals are distributed in the lumbo-sacral plexus, and those from the proximal lower limb are distributed in the lumbar plexus. Those from the upper extremities travel through the brachial plexus.

Sensory fibers next traverse the neural foramen of the spinal column and enter the spinal canal. Ultimately these fibers transition into the CNS and join other fibers in the tracts of the spinal cord and/or terminate intra-segmentally at synapses in spinal cord gray matter. The distance from the site of the neural foramen at which an individual fiber enters the spinal column and the site at which it enters the spinal cord decreases caudo-cranially. Fibers from the lower extremity and genitals have the largest distance to traverse in this sense, as they enter the lower levels of the spinal column and ascend the cauda equina before reaching their respective entry points of the lumbo-sacral enlargement and the conus medullaris at approximately the L1 vertebral level. Conversely, fibers from the upper extremity enter the spinal column at near equivalence with the spinal cord segmental level.

Sensory fibers from a given nerve do not simply enter the spinal column all at the same level. Rather, fibers from the nerve enter several adjacent spinal levels. Correspondingly, these fibers enter the spinal cord through dorsal nerve roots at the dorsal root entry zone at several segmental levels. Once in the spinal cord, sensory fibers ascend via multiple parallel pathways. The general consensus is that the dorsal or posterior column spinal pathways [5, 59–62] primarily mediate the SEPs. It has been suggested that other pathways such as the dorsal spinocerebellar tracts (which actually are lateral and lie over the lateral corticospinal tract) [63, 64], and the anterolateral columns [65] may contribute to the early SEP responses that are used for monitoring purposes. However, it also has been suggested that the SEPs elicited by electrical peripheral nerve stimulation with latencies less than 100 ms are selectively mediated by the dorsal somatosensory system because the abundant thick peripheral axons which mediate these responses have low thresholds and fast, uniform conduction. SEPs are thought to not be mediated by the anterolateral system because this system consists of thinner axons which have higher thresholds and slower, more variable conduction velocities [22]. What remains controversial is the traditional view that the proprioceptive afferents which mediate the SEPs only ascend the dorsal columns and directly project to the cortex. There is some evidence that indirect pathways may exist, the so called postsynaptic dorsal column pathway, and play a role in conveying proprioceptive information to the cortex as well [22]. The anatomy of the cutaneous and proprioceptive contributions to the signal remains an important consideration when interpreting SEPs.

Upon entering the spinal cord, the axons that comprise the dorsal columns remain ipsilateral to the side of the hemi-body they represent. They distribute topographically within the dorsal columns such that axons corresponding to lower extremity and genitals border the dorsal median sulcus, with axons from the trunk and then the upper extremity systematically populating the dorsal columns laterally. The dorsal columns on each side of the midline are further distinguished by the dorsal intermediate sulcus into medial and lateral fascicles—the fasciculus gracilis which corresponds roughly to the lower extremities and the fasciculus cuneatus which corresponds roughly to the upper extremities.

Sensory fibers from the dorsal columns enter and ascend the dorsal aspect of the lower brainstem before synapsing ipsilaterally in the dorsal column nuclei of the lower medulla. For those fibers which directly ascend the posterior columns of the spinal cord, this is the first synapse in the pathway and neurons of the dorsal column nuclei are often defined as second order.

Fibers originating from the fasciculus gracilis and the fasciculus cuneatus terminate in the nucleus gracilis and the nucleus cuneatus, respectively, thus grossly maintaining the anatomical division of the lower and upper extremities established in the spinal cord. At the upper boundary of the pyramidal decussation of motor fibers in the medulla, projections from neurons of the dorsal column nuclei decussate as the internal arcuate fiber tract. A small but notable portion of the general population has an uncrossed sensory pathway, due to the abnormal absence of the internal arcuate. Relevant to IONM, this includes patients with horizontal gaze palsy and progressive scoliosis. In these patients, the clinician should check for decussation when developing the monitoring strategy and obtaining the patient’s baseline. As projections from the dorsal column nuclei decussate, they will position themselves more anteromedially and ultimately ascend on the contralateral side of the brainstem, forming the dense fiber bundle called the medial lemniscus.

Medial lemniscus fibers terminate in the thalamus, synapsing with neurons of the ventral posterolateral nucleus (VPL) and other components of the ventral posterior complex. This is the second synapse in the pathway that directly mediates the SEP, and these thalamic neurons are often defined as third order. Fibers arising from the nucleus gracilis terminate lateral to those of the nucleus cuneatus, within the VPL. Thalamic afferents traverse the posterior limb of the internal capsule on their way to the cerebral cortex.

Thalamocortical projections that mediate SEPs fan out in the cortical radiations and terminate in the primary sensory cortex (S1), the locus of which is predominantly in the postcentral gyrus of the parietal lobe. The topography appreciated thus far throughout the dorsal column—medial lemniscus pathway is reflected in a somatotopically organized S1. The somatotopy is also homuncular, meaning that richly innervated structures such as the hands, feet, face, and lips have a disproportionately greater cortical representation. Deep within the interhemispheric bank is the sensory representation of the genitals and the pudendal nerve. The representation of the foot is just superior to that as are the representation of the distal nerves of the lower extremity. Starting around the cortical apex, the leg, trunk, head and neck, shoulder, arm, distal arm, and hand are systematically distributed from medial to lateral. Correspondingly, the representation of the nerves of the proximal lower extremity is near the cortical vertex whereas the representation of nerves of the distal upper extremity is lateral. The face and lips, the tongue, throat, and pharynx, as well as the representation of nerves of the face and throat are represented most laterally.

### Blood supply

Normal functional status of those nervous system components which mediate the SEP depends upon the blood supply and the specific arterial branches which provide this supply. While peripheral nerves like the ones that are stimulated to elicit SEPs may be less susceptible to ischemia than other portions of the SEP pathway, it remains important to consider the circulatory anatomy that supplies them. Select peripheral vessels have specific relevance, since they are common sites where vascular compromise with associated changes in perfusion results in functional changes in the SEP. In the lower extremity, the distal portions of the leg are supplied by the popliteal artery, which is fed by the femoral artery which, in turn, is fed by the external iliac artery. The external iliac artery is at risk of retractor-dependent compression and reduced perfusion during anterior approaches to the lumbar spine. This can result in changes in conduction in the nerves of the leg, and suppression of lower extremity SEPs [66–68]. Lower leg ischemia including femoral artery ischemia can also result from patient malpositioning [67, 69]. In the upper extremity, the distal portions of the arm are supplied by the radial and ulnar arteries which are fed by the brachial artery which, in turn, is fed by the axillary artery. The brachial and axillary arteries are common sites of patient position-dependent compression and reduced perfusion due to unsuitable positioning of the patient. This can result in changes in conduction in the nerves of the arm and suppression of upper extremity SEPs [67, 70, 71].

The blood supply in the spinal cord responsible for perfusing the dorsal column pathways which mediate SEPs is generally thought to originate from the longitudinal posterior spinal arteries and perforating branches of the arterial vasocorona [55–57, 72]. The anterior spinal artery is generally believed to provide the primary blood supply to the anterior and antero-lateral portions of the spinal cord, which make up the remaining two-thirds of the spinal cord. Both the anterior and posterior spinal arteries receive their blood supply from the aorta. In the cephalad region, they are fed by the vertebral arteries. However, as the spinal arteries descend along the spinal cord, they receive segmental perforators from the aorta. Whereas the paired posterior spinal arteries receive blood flow via small radicular arteries at most vertebral levels, the anterior spinal artery receives its blood flow from only two to eight radicular arteries [73, 74]. In particular, the thoracic spinal cord usually has only one to three anterior segmental arteries arising from the aorta. As a result, it is particularly susceptible to ischemia. Blood flow to the spinal cord can be compromised by reductions in blood pressure due to the relatively long distances between major blood vessels and the region between the thoracic levels T4 and T7 is considered to be the least well-supplied region of the spinal cord. One anterior segmental artery is larger than the others and supplies about 75% of the blood flow to the anterior spinal artery. This artery is known as the Artery of Adamkiewicz also referred to as the arteria radicularis magna or the great anterior radiculomedullary artery. Motor pathway function is mediated by spinal cord pathways which receive their blood supply from this artery [74]. Loss of motor function due to compromise of the blood supply to the anterior spinal artery may be associated with little or no loss of sensory function, which is mediated by the dorsal column pathways (a condition known as anterior cord syndrome [75]). However, the degree to which this is true is uncertain and may vary between individuals.

Blood supply to the portions of the brainstem that mediate the SEP is from diverse sources [56]. The portion of the lower medulla where the dorsal column nuclei are located is perfused by the posterior spinal artery, whereas the portion of the medulla where the lower medial lemniscus is located is perfused by the anterior spinal artery. At higher levels, the location within the medulla through which the medial lemniscus courses is nourished by branches of the vertebral arteries and paramedian branches of the caudal basilar artery. The portion of the pons through which the medial lemniscus courses receives blood supply via the paramedian and long circumferential branches of the basilar artery. In the midbrain, the medial lemniscus receives blood supply from the posterior cerebral and the superior cerebellar arteries.

The ventroposterior complex including the VPL of the thalamus receives blood supply primarily from thalamogeniculate branches of the posterior cerebral artery. Blood supply to the posterior limb of the internal capsule arises from the middle cerebral artery, particularly the lenticulostriate branches and to a lesser extent the anterior choroidal artery.

The blood supply to S1 originates primarily from two sources. The middle cerebral artery provides the blood supply to the lateral area of the cortex and subcortical white matter which mediates the upper extremity SEPs whereas the anterior cerebral artery provides the blood supply to the medial area of the brain and subcortical white matter which mediates the lower extremity and pudendal SEPs.

## Methodologies

Early guidelines attempted to address the technical requirements of instrumentation used to acquire SEPs in order to provide safe and effective monitoring capabilities [12, 13, 15]. Those requirements have continued to evolve [18–21].

### Equipment standards

The instrumentation used to acquire SEPs consists of a set of amplifiers. The basic amplifier, known as a single ended amplifier, consists of three terminals or contacts; an input, an output, and a ground contact. When recording electrical activity, the voltages that are present at the input and output of the amplifier are measured relative to, or referenced to, the ground. The gain of the amplifier is the amount a signal or input voltage is amplified. It is the ratio of the output voltage divided by the input voltage. If in addition to increasing the amplitude of the input signal, the amplifier also inverts its polarity, it is known as an inverting amplifier. If two single-ended amplifiers with the same gain are connected together, one inverting and the other non-inverting, the result is a differential amplifier. The resulting differential amplifier now has two inputs, one output, and a reference ground. Ideally, a differential amplifier amplifies only the voltage difference between the two inputs and any signal that is common to both inputs is totally rejected. This result is known as common mode rejection. In reality, the signal common to both inputs is never completely cancelled out because of small differences in the gains of both differential inputs and is therefore present in the output signal to a small degree. The resulting ratio of the size of the amplified input signal difference (the differential gain) to the gain of the signal common to both inputs (common mode gain) is known as the common mode rejection ratio or CMRR [76].

An amplifier’s dynamic range is the span of input voltages over which the output voltage is proportional to the input. Outside the dynamic range of an amplifier, the output cannot follow the input. If the input voltage is less than the minimum voltage in the range, the output voltage will be zero. On the other hand, if the input voltage is larger than the maximum value in the range, the amplifier’s output will saturate, and the signals will be clipped and distorted [76]. Therefore, the recommendation is to make sure the input voltage is within the amplifiers’ dynamic range so as to contain unclipped biologic signals.

The signal-to-noise ratio of acquired electrophysiological activity refers to the proportion of the amplitude of the signal to the amplitude of the background interference or noise in that activity. Single recorded trials resulting from a single stimulus contain a combination of electrophysiological signals as well as electrical artifact or noise. The amplitude of these trials is a function of the amplifier’s sensitivity. One way to improve the signal-to-noise ratio is to adjust this sensitivity. The sensitivity of an amplifier determines what input values cover its entire dynamic range. If the sensitivity is very high, a small input voltage will result in a large output signal. If recorded activity exceeds a certain voltage level and can safely be considered to be of non-electrophysiological origin, it should be rejected and single trial activity which exceeds a certain amplitude can be programmed to do so. If the sensitivity of the amplifier is set too high, a large number of responses containing signals are rejected, whereas if it is set too low, the recordings are contaminated with large amounts of noise or artifacts. Therefore, sensitivities, or rejection levels, should be set to pass biological signals and to exclude higher amplitude artifacts so as to avoid excessive rejections that delay acquisition [50].

For processing purposes, the amplitudes of the recorded analog signals are converted to a series of digital samples by sampling the analog signals at a fixed rate. For accuracy purposes, each amplifier channel should be capable of at least sixteen-bit digital resolution [22, 50]. In addition, the digital sampling rate should be more than twice the highest frequency content of the sampled analog signal in order to prevent aliasing. Therefore, a sampling rate of 3–4 kHz would be sufficient for recording biological signals consisting of frequencies less than 1 kHz [22, 50].

### Technical parameters

#### Number of channels

Based on the requisite number of recording sites needed for monitoring the responses from each stimulation site and the need to interleave stimuli between multiple stimulation sites, it is recommended that IONM machines have at least six recording channels for each monitoring modality (upper or lower extremity SEPs); three for each extremity. Six channels will allow for simultaneous display of one channel each of cortical, subcortical, and peripheral responses from a pair of extremities. If the responses from more than one monitoring modality are simultaneously acquired (i.e., SEPs and spontaneous EMG), additional recording channels may be necessary and equipment requirements must be adjusted accordingly [20, 21].

#### Filters

In IONM, the choice of filter settings plays a critical role in optimizing the acquisition of responses. The main objective is to obtain responses that are easily interpretable and that require minimal averaging in the shortest time possible. Unlike routine laboratory testing, where filters are typically set at 20 Hz–3 kHz and kept constant from one patient to another, IONM requires different filter settings that are optimized for the highly electrically noisy environment of the operating room (OR) and may vary from one patient to another.

These OR-optimized filter settings are set at the beginning of the surgery and are not changed during the procedure unless it is absolutely necessary to do so. If the choice is made to revise the filter setting during the procedure, it is important to document those changes due to their effect on the signal morphology and for accurate interpretation of the results.

The recommended filter settings for intraoperative SEP signal acquisition parameters evolved from their use in the diagnostic setting. However, the various waveform morphology subtleties that these filter settings are important for in a diagnostic setting are far less important in the intraoperative monitoring setting. This is because, in IONM, it is the changes in waveform morphology that are important.

It has been suggested that for cortical responses, the system band-pass should be initially set to 1–30 to 250–1000 Hz [12, 13, 15] while for subcortical responses, the system band-pass should be 30–100 to 1000–3000 Hz [12, 13, 15]. The relative frequency content of cortical responses is lower than that of subcortical responses, with the majority of the energy contained in cortical SEP responses present in the frequency pass band above 30 Hz and below 500 Hz. Hence, to record these responses, it is often useful to set the high frequency filter to as low as 300–500 Hz to eliminate unwanted high-frequency signals. Increasing the high frequency filter settings to greater than these will have very little effect on the physiological frequency content of the intraoperative evoked responses and will only increase the amount of high frequency environmental noise that is recorded. Suitable low-high filter settings for scalp recordings would be 30–300 Hz [22, 50]. On the other hand, peripheral nerve responses consist of significant high frequency components. Therefore, suitable filter settings for acquiring cubital and popliteal fossa responses would be 0.2–1000 Hz [22, 33, 50].

In an electrically hostile environment like the OR, many of the pieces of equipment used during surgery produce electrical signals that can contaminate the neurophysiological responses with signals both in the low and high frequency ranges. Therefore, it is not uncommon to set narrow recording band-passes to avoid the acquisition of excess artifact from these sources. Widening the pass band so that it includes more high and low frequency activity is likely to also invite the inclusion of unwanted environmental noise, including 60 Hz noise from devices and electrical power sources in the OR.

Although 60 Hz artifact is common in the OR environment, the 60 Hz notch filter is not recommended because of the “ringing” artifact it can cause resulting in distortion of the recorded responses. Its utilization should be limited to a last resort when useful responses cannot be acquired without it.

#### Time bases

The time bases used to acquire and display responses from the upper and lower extremities should account for the normal conduction time between the stimulation and recording sites. This of course will depend upon factors such as the age and height of the individual and the presence of any pathological conditions that result in slowed neural conduction. When elicited at the wrist, the latency of the peak of the upper extremity cortical response which is typically used for monitoring purposes normally appears about 20 ms (msec) after the stimulus onset. For the lower extremities, when elicited at the ankle, the cortical peak of interest normally appears at twice this latency or at around 40 ms after the stimulus onset. Therefore, the time bases for upper and lower extremity responses are generally set at about 50 and 100 ms, respectively. However, the time bases may be adjusted to optimize the acquisition and display of the individual patient’s waveforms. Most commonly, this means increasing the time base to be able to capture delayed responses.

### Set-up procedures

Monitoring equipment set-up in the OR should largely be completed prior to the patient’s arrival to the OR suite. Ideally, the equipment is placed in a location in the OR that is close enough to the operating table to permit easy dialogue with both the surgeon and the anesthesiologist and to allow observation of the surgical procedure. The stimulation and recording modules should be attached to the OR table so that they are conveniently located for easily attaching stimulation and recording electrodes and their cables as well as to access them for troubleshooting purposes during the procedure. The modules and cables should be properly secured so that they do not result in any safety hazards and are safe from fluid spills. The appropriate monitoring modality software should be loaded in anticipation of acquiring baseline data prior to surgical incision. In certain cases, such as those involving patients with spinal instability, it is prudent to collect baselines prior to patient positioning.

Ideally, if time constraints permit, interaction with the patient and the anesthesiologist to discuss the surgical plan, can occur prior to the patient being taken to the OR. This would ensue in the holding area and would permit the anesthesiologist to inform the patient regarding the planned use of monitoring during their surgical procedure and to address any questions or concerns. In addition, if adhesive surface electrodes are to be used for monitoring, these electrodes can be preoperatively applied and secured at the bedside thus shortening the OR setup and enabling early post-induction recording and signal optimization. Similarly, the preoperative setting provides a good opportunity to measure and mark the scalp for the most accurate electrode placement.

### Patient preparation

#### Stimulation electrodes

Optimal SEP monitoring is dependent upon consistent and reliable stimulation throughout the surgical procedure. Several types of electrodes can be used for stimulation purposes. These include bar electrodes, EEG metal cup disc electrodes, and disposable adhesive surface and subdermal needle electrodes. All can be effectively used but each has its advantages and disadvantages. Bar electrodes and metal cup disc electrodes are used in conjunction with electrode paste and are reusable. The adhesive surface electrodes utilize an integrated conductive gel. Although electrode pastes and adhesive gels may dry out or change their electrical conductance characteristics during lengthy surgical procedures, the use of constant current stimuli will compensate for any change in electrical conductivity if the electrodes remain securely in place. The non-disposable rigid bar electrode is susceptible to being displaced and so may produce erratic responses in the OR if it is not well secured. In addition, their use is not advised because of the risk of sustained-pressure skin necrosis [50]. EEG metal cup disc electrodes are more difficult to secure than either the subdermal or adhesive surface electrodes. Hence metal cup disc and bar electrodes are now rarely used intraoperatively. The electrodes of choice are generally disposable and may include either adhesive surface and/or subdermal needle electrodes. Subdermal electrodes are invasive, they are associated with concerns regarding infections and/or bleeding and must be handled with care to avoid inadvertent needle sticks [50, 76]. In addition, in cases when the electrosurgical cautery device is not properly grounded, tissue burns may occur at the insertion site of needle electrodes because of their small surface area and the high current densities that result [50, 76]. Despite these concerns, they are routinely used for recording and stimulation purposes [77]. In addition, if the patient has severe edema, swollen wrists or ankles, or preexisting neurological deficits, the use of subdermal needle electrodes may be the only option for stimulation.

#### Stimulation sites

The choice of what nerves to stimulate will largely be dictated by the location of the surgical site. SEPs are typically elicited by stimulating either the median or ulnar nerves in the upper extremities or the tibial (posterior tibial) or peroneal nerves in the lower extremities. Stimulation sites are generally chosen because of easily identifiable anatomical landmarks and the ease with which a stimulating electrode can be placed near the nerve to be stimulated. Unless the sites are unavailable, upper extremity stimulation electrodes are normally placed near the wrist. To stimulate the median nerve, the cathode of the stimulating pair of electrodes should be placed about 2–4 cm (cm) proximal to the wrist crease between the tendons of the palmaris longus and flexor carpi radialis muscles. The anode electrode should be placed 2–3 cm distal to the cathode. Similarly, for ulnar nerve stimulation, the cathodal electrode should be placed 2–4 cm proximal to the wrist crease on either side of the tendon of the flexor carpi ulnaris muscle and the anode should be placed 2–3 cm distal to the cathode [12, 13, 15, 78]. If these sites are inaccessible or if the SEPs elicited by their stimulation are unmonitorable, SEPs can be activated at alternate sites. For upper extremity SEPs, a common alternate stimulation site is the ulnar cubital notch, and a less common alternate stimulation site is the antecubital fossa. These alternate sites activate the ulnar and median nerve, respectively. For proximal ulnar nerve stimulation, electrodes are placed at the medial epicondyle of the humerus arranged with a cathode over the humerus and an anode 2–3 cm distal [79].

If the median and ulnar nerves at the wrist are unavailable or for other reasons, another effective site of stimulation in the upper extremity is the superficial radial nerve at the wrist [80, 81]. In addition, locations on or near Erb’s point (EP) for non-specific activation of the brachial plexus may be considered. Stimulation of individual digits may be appropriate in specific circumstances. Specific considerations for the intraoperative use of these alternate sites are not well documented.

In order to acquire SEP responses from the lower extremities, stimulation of the tibial nerve is normally done near the ankle and stimulation of the peroneal (fibular) nerve is normally done slightly distal to the knee near the head of the fibula. To stimulate the tibial nerve, the cathode should be placed between the medial malleolus of the ankle and the Achilles tendon just proximal to the malleolus. The anode electrode should be placed 2–3 cm distal to the cathode or inferior to the prominence of the malleolus. This placement overlies the nerve as it follows a path around the malleolus. To stimulate the peroneal nerve, the cathode should be placed distal to the lateral aspect of the knee and slightly medial to the head of the fibula and the anode electrode should be placed 2–3 cm distal to the cathode [12, 13, 15]. For lower extremity SEPs, a common alternate site is the popliteal fossa, the stimulation of which is not clear whether it produces a non-specific activation of either or both the tibial and the fibular branches of the sciatic nerve. To place this electrode pair, palpation of the space behind the knee is performed to find the point at which the medial and lateral gastrocnemius muscles split. The anode is placed near this location, just below the skin crease at the back of the knee and near the lateral to medial midpoint. Then the cathode is placed 2 cm above the anode.

For certain applications, SEPs are elicited at other anatomic loci, and often in addition to the sites indicated above. For SEPs of the lower extremity, these include the top of the foot to activate the distal branches of the peroneal nerve [82], the medial thigh or lower leg distal to the knee on the tibial surface to activate the saphenous nerve, and the inguinal crease to activate the trunk of the femoral nerve [83–85].

Non-limb SEP stimulation sites include the penis and clitoris to activate the penile and clitoral nerves, respectively, for monitoring pudendal nerve SEPs [86]. Finally, for cranial nerve SEPs, stimulation sites include the gums or tongue for activation of the gingival and/or lingual distal branches of the trigeminal nerve [87, 88].

For monitoring purposes, it is extremely important to select nerves whose responses are mediated by neural tissue at risk during surgery. Therefore, when the thoracic region of the spinal cord is at risk, monitoring median nerve responses to detect an iatrogenic spinal cord insult would be useless whereas monitoring tibial nerve responses would not. It is best to choose to monitor the responses of nerves which are entirely mediated by tissue located below the area at risk [12, 13]. In addition, the choice of what nerves to stimulate may also result from other factors such as what neurological structures are at risk as a result of positioning, which nerves are accessible or which nerves, when stimulated, will simply provide the best responses. For example, changes in lower brachial plexus function due to positioning are generally best detected by monitoring ulnar rather than median nerve function [71, 89–93]. In patients with large edematous legs, proximal fibular nerve stimulation may provide better responses than tibial nerve stimulation. For more information regarding the choices of nerves to stimulate for IONM during various surgical procedures, see Sect. 7.

#### Recording electrodes

Just as it was important that the stimulation electrodes be associated with a consistent and reliable stimulus presented in a safe manner, it is also important that the recording electrodes provide consistent, reliable, and good quality recordings in a safe manner as well. Low impedance and electrode lead twisting or braiding are important for reducing extrinsic electromagnetic interference [22, 50]. Subdermal needle electrodes should measure less than 5 kOhms impedance to minimize noise and optimize recording [22].

Subdermal needle or metal surface “cup” electrodes (gold, silver, or tin) are typically used for recording from the body surface [15]. The subdermal needle electrodes are convenient to use because they can be quickly and easily placed. However, if they are not taped or fastened down, they can be easily displaced; usually either during patient positioning, by the anesthesiology team member reaching under the surgical drapes, or while preparing to take an x-ray. Alternatively, a corkscrew version of the straight subdermal needles or surface electrodes can be used instead. Corkscrew electrodes are literally screwed into the scalp and are difficult to displace. Care must be exercised not to over-tighten the corkscrews as this can have the unwanted result of excessive tissue damage under the electrode.

A “strip” or grid electrode array can be used for direct cortical recordings of SEPs. These types of recordings are used for correlating structural and functional anatomy (electrocorticography) [45, 46].

#### Recording sites

Because the goals of IONM are different than those in the diagnostic laboratory, the recording montage used for IONM purposes may be different than those for diagnostic use. The recording montage will depend upon the number of recording channels available. It may also depend upon whether responses can be simultaneously recorded from both sides of the body and whether replication is desired. The basic principle of mixed nerve SEP monitoring is to stimulate distal to the surgical site at risk and to record at a site(s) proximal to the surgical site. In most cases, these recording sites should include at least one cortical and one subcortical recording site. An additional peripheral recording site can be placed proximal to the stimulating site but distal to the surgical site. It is of value to record cortical responses in all cases since they provide an indication of the functional integrity of the entire pathway, anesthetic management, and because they are readily recognized. However, reliance only on the cortical responses can result in false positive changes because they are significantly affected by general anesthesia. Because fewer synapses are associated with mediating the subcortical response, anesthetic effects are less pronounced than on the cortical responses. However, reliance on only subcortical responses during spine surgery can also result in false positive findings due to the quality of the subcortical responses, their generator sites, and other factors. Of note, it has recently been suggested that only cortical and peripheral responses be acquired because of the low signal to noise ratio of subcortical responses and the resultant additional time needed to acquire these responses [22, 50].

Peripheral recording sites are distal to tissues that are at risk of iatrogenic injury and serve as a critical first point of evaluation of the SEP. Their main purpose is to verify the stimulation and that the pathway of interest is being activated consistently throughout the procedure. They help to delineate the site of a conduction block that has been detected in the proximal recordings. They may also serve as key monitoring loci in specific instances such as peripheral nerve and non-spine orthopedic surgeries.

Normally each peripheral recording site corresponds to a stimulated limb. Therefore, peripheral sites for upper extremity are distinct from those for lower extremity, unlike several other later components of SEP recordings. For upper extremity SEPs, the most common location for peripheral recordings is near Erb’s point (EP), in the space 1–2 cm above the clavicle and between the tendons of the sternocleidomastoid and the trapezius muscles. Alternatively, recording electrodes are placed below the clavicle or in the axilla. A proximal peripheral site has also been recommended; the antecubital fossa, located anterior to the elbow as a small depression at the junction of the arm and the forearm [94]. For lower extremity SEPs, the most common location for peripheral recordings is at the popliteal fossa (PF) in the space behind the knee, superior to the top of the gastrocnemius muscle and midway between the tendons of the hamstring muscles.

Subcortical recordings often employ the same locations as those used to obtain cervical segmental recordings. Segmental recordings are also commonly referred to as cervical and lumbar for upper and lower extremity SEPs due to the location of their respective recording sites. A common cervical segmental recording location is the midline in the back of the neck over the spinous process of the fifth cervical vertebrae (CS5), but an electrode may also be placed midline over other cervical vertebrae or the inion. If the back of the neck is inaccessible, an electrode placed midline on the front of the neck (anterior cervical, AC) or laterally over the mastoid or tragus, either both mastoids or tragi (either independently or electrically linked) may be considered. Lumbar segmental recording sites are fewer and are placed midline in the lower back over the T12—L1 vertebrae.

The cortical recording site in or on the scalp is used to record the SEP as it arrives at its endpoint in the postcentral gyrus of the contralateral somatosensory cortex. Generally, the location of the electrodes for upper extremity stimulation is at CP3 or CP4, contralateral to the side of stimulation and 10% posterior to the C3 and C4 positions of the 10–20 International, and the 10–10 modified Systems of EEG electrode placement [95]. For lower extremity stimulation, the cortical recording site is at CPz, on the midline and 10% posterior to the Cz position of the 10–20 International and 10–10 modified systems of electrode placement although the second cortical recording can be placed at CPi due to paradoxical lateralization [95].

To reduce the amount of noise pickup, the ground electrode is best placed distal to the first recording site, such as the forearm or leg [13, 96]. Multiple reference grounds were never used in older machines with isolated grounds because they introduced ground loops which may introduce excess noise in the recordings. Newer machines do allow for the use of multiple reference grounds. However, currently it is more common to use a single ground electrode; the location of which varies from practice to practice. An earth ground should never be used for safety reasons because it provides an alternate path for the surgical electrocautery current. Keeping the recording input leads short and the electrode impedance values at 2.5 kOhms or lower for gold disc or subdermal electrodes will help to minimize the amount of stimulus artifact and other electrical noise that is recorded. However, the acquisition of some stimulus artifact can be useful because it demonstrates that the stimulators are functional when troubleshooting is necessary.

### Data acquisition

#### Stimulation technique

SEPs are elicited by electrical stimulation of a peripheral nerve at a distal site. There are various ways of presenting the electrical stimuli in order to elicit SEPs. The earliest versions of monitoring equipment only allowed the responses to be recorded from stimulation at a single site. For validation purposes, this acquisition process was then repeated to ensure that the responses replicated. A similar set of responses were then acquired from the opposite extremity, and it would typically be several minutes before a new set of responses could be acquired from the first stimulation site. This format significantly delayed the detection of a unilateral SEP change.

Improvements in the data acquisition equipment have occurred which make it possible for stimuli to be interleaved between a pair of extremities such that the responses from each extremity are essentially being recorded simultaneously. Left–right alternating and interleaving reduces acquisition time by one half and enables concurrent bilateral recording. Interleaving the responses from four limbs also reduces acquisition by one half but may not further improve acquisition time to accommodate the acquisition of four different responses. Nevertheless, this technique enhances cortical SEP amplitude due to the slower presentation of the stimuli [22, 97]. This improvement has been widely adopted, has resulted in faster data acquisition, and has permitted the rapid determination of SEP changes and side-to-side asymmetries [12, 13, 15, 98].

In general, stimulation is applied at locations where a given nerve courses close to the surface of the skin or where it is otherwise accessible being relatively unobscured by other tissues. Not incidentally, many of these sites are also amenable to capturing distal nerve action potential volleys, either orthodromic or antidromic, as a part of the recording strategy of SEPs and other monitoring and mapping modalities. Depending on the location of stimulation, transmission in the nerve may result directly from stimulus-dependent activation, indirectly subsequent to the activation of distal cutaneous receptors or some combination of the two. Because of the potential for stimulus “spread”, the clinician must also know the anatomy around the site of stimulation since nerves adjacent to the nerve of choice at that site may be inadvertently activated which has important implications on the SEP. Such activation is known as “current jumping” when an adjacent nerve is unintentionally activated. High stimulus intensity or long pulse duration may be necessary to drive recruitment in the preferred nerve but also make the unwanted and confounding activation of adjacent nerves more likely whereas low stimulus amplitude and short pulse duration increase specificity albeit while decreasing recruitment [99].

When using surface versus subdermal needle electrodes to deliver the stimuli, a proximal cathode and a distal anode should be spaced about 2–3 cm apart. Current spread to the underlying nerves is the effective stimulus and the use of a constant current stimulus is meant to compensate for any changes in contact resistance. However, the intensity of the constant current stimulus and the ability to compensate for contact resistance changes are limited by the maximum output voltage of the stimulator. When the contact resistance is excessive, the output of the stimulator will be current limited. Most machines designed for the purposes of acquiring evoked potentials will indicate a warning when this is the case. Use of a constant voltage stimulus provides a constant stimulus intensity only if the contact resistance does not change. For this reason, the use of constant current stimulation is recommended [12, 13, 50].

An electrical stimulus is typically presented as a succession of rectangular pulses with a certain amplitude, pulse duration and frequency of presentation. The intensity of the stimulus is dependent on its amplitude, pulse duration and frequency. An increase in any of these parameters will normally cause an increase in stimulus intensity because the amount of current flow or delivered charge will increase [100]. However, the way the underlying nerves or tissue reacts to the stimulus is not solely dependent on the stimulus intensity but is also dependent upon the placement of the stimulation electrodes in relation to the intended neural structures to be activated. For some patients with large or edematous extremities, the current spread resulting from the use of surface electrodes may be ineffectual for exciting the intended underlying neural structures. In such cases, the use of subdermal needle electrodes may be more effective. Subdermal needle electrodes can be placed closer to underlying nerves than surface electrodes. As a result, the stimulation intensities needed to stimulate underlying nerves will be less when using subdermal needle electrodes rather than surface electrodes. It is suggested that a pulse duration of 200–300 microsecond be used for eliciting SEPs [12, 13, 22, 50]. Controlling the stimulus rate is essential in obtaining high quality evoked responses. The critical factor in obtaining evoked responses is the assumption that the response and the underlying noise are not synchronized. Thus, to have the noise decrease in amplitude with averaging, the stimulus rate should not be a submultiple of any noise frequency. As the most common noise frequency is 60 Hz, it is important that stimulation rates that harmonize with 60 Hz such as 4.0, 5.0, or 10.0 Hz not be used [12, 13, 15]. Often, there are other sources of noise in the evoked response and sometimes minimally changing the stimulus rate (for example from 4.7 to 4.9 Hz) may change the quality of the recorded evoked potentials in the setting of high amplitude rhythmic noise [101]. Many contemporary IONM machines have a function to evaluate the noise profile which can assist in selecting the optimal stimulus rate(s). Stimulation rates between 2 and 5 Hz are recommended [12, 13, 15, 22, 50]. However, lower stimulation rates (between 1.5 and 3 Hz) can sometimes improve poor lower extremity responses [50], particularly when compromise of neurological function (e.g., neuropathy) is present, whereas upper extremity SEPs may demonstrate little or no change at stimulation rates as high as 9 Hz. Increasing the stimulus rate beyond 9 Hz for the upper extremity SEPs and 5 Hz for the lower extremity typically results in a substantial degradation of the SEPs, particularly the cortical responses [12, 13, 15]. It has been reported that the best stimulation rate for acquiring single median nerve SEP recordings is 12.7 Hz but this is only true for recording periods less than or equal to 5 s. Otherwise, the best stimulation rate for acquiring either median nerves or tibial nerves is around 4.7 Hz [102]**.**

Supramaximal stimulation intensities are safe and should be utilized to produce repeatable responses and ensure that variations in response amplitudes are not a result of variations in effective stimulation intensities [50]. Generally, it should not be necessary to utilize stimulation intensities which exceed 50 milliamps (mA) to elicit repeatable SEPs and to provide effective monitoring [15]. Although commercial stimulators can generally provide stimulation intensities greater than 50 mA, it is unusual for a stimulus of this intensity to be ineffective for eliciting SEP responses unless pathology is present or the current from the stimulating electrode is not reaching the underlying neural tissue at a sufficient intensity to cause excitation. In such cases, current shunting may be occurring. Current shunting provides an alternate low-resistance pathway(s) for electrical current to flow or disperse.

The effectiveness of the stimulus for eliciting well-defined, repeatable responses will vary between patients and will depend on several factors including (a) the type of stimulation electrodes being used, (b) the proximity of the electrodes to the underlying neural structures, (c) the anesthetic management, and (d) certain patient comorbidities (e.g. diabetic peripheral neuropathy) and the conduction status of the neural pathways being monitored. The person providing the monitoring should consider options such as optimizing the stimulation electrode placement, changing to subdermal needle rather than surface stimulation electrodes, or selecting an alternate stimulation site. Increasing stimulus intensities to as high as 100 mA may be necessary to produce an effective stimulus. Although concerns may exist regarding the possibility of tissue damage resulting from high current densities at the stimulation sites, these concerns appear to be unfounded and others have indicated that there is no evidence in the literature or otherwise to support them if the stimulus parameters available on commercially available devices are utilized [22]. The value of the use of SEPs in intraoperative neuromonitoring is proportional to the frequency of its acquisition. It is generally understood that continuous SEP stimulation and acquisition is a best practice in most operative scenarios [50, 97].

#### Recording technique

The acquisition of intraoperative SEPs is based on certain objectives, some of which relate to the recording technique. These objectives incorporate the following: the value of the data at a given recording site relative to the risks of the surgery or those of peri-surgical events, the relative likelihood of collecting a signal at a given recording location in a reasonable time frame, and the overall value of knowing details of the conduction throughout the pathway versus knowing that the signal has reached some critical end point. An important aim of intraoperative SEPs is to sample the activity of discrete, critical loci of the pathway via strategically placed recording electrodes [19, 21], not dissimilar to diagnostic SEPs in the clinic. Multiple sampling sites along the pathway can support and focus localization efforts, neurophysiologically, when a discrete source of a conduction block needs to be elucidated. For example, if there are samples being taken of peripheral, subcortical and cortical potentials and the initially well-formed potentials are suddenly absent from the cortical sites alone with preservation of the subcortical and peripheral recordings, one’s focus is on a possible conduction block between the subcortical and cortical generators or at the level of the cortex itself. Multiple sampling locations also has trouble-shooting benefits since, for example, the sensitivity to anesthetic and systemic factors of peripheral versus deep brain versus cortical locations varies [103]. One can use the information from potentials collected at multiple sampling locations to differentiate problems with the functional integrity of the dorsal column and medial lemniscus pathways from the impact of these other factors. Additional recording sites or recording derivations may help to distinguish perfusion territories or tissues with generator sources that are temporally or spatially close. Sampling across multiple locations of the pathway while acquiring intraoperative SEPs is counter-balanced by certain realities. For example, given the short time allowed for patient set-up and also for acquisition of reliable baselines as is common in the OR, the value of certain recording sites may not balance the additional time required to place them. While generally not a problem when implementing an SEPs-only protocol, as they are quick and easy to administer on their own, this may be an issue given that they are often incorporated in monitoring protocols with additional modalities whose set-up is burdensome.

In some instances, the critical recording site is distal, such as the peripheral potential monitoring site used to protect against nerve damage during hip surgery [104, 105]. Therefore, the protocol does not require that samples be taken from the proximal pathway such as the cortical potentials. On the other hand, those recording sites that capture cortical potentials are the minimum necessary to assess functional continuity of the entire pathway and function at this end point. Some argue that for many applications of intraoperative SEPs, the cortical recording sites are largely sufficient and that other components of the pathway are either less or not relevant, distracting, poorly recordable or obscure, and/or may be elucidated via other means [22, 50].

#### Averaging

The evoked electrophysiological activity that contributes to the recorded responses is usually only a fraction as large as the background random noise activity in which it is buried. Averaging is the standard procedure for eliminating this background random noise activity whose amplitude decreases in proportion to the square root of the number of trials contributing to the average [22, 94, 106]. However, averaging is not effective for eliminating non-random noise from many devices utilized in surgery such as image intensifiers (fluoroscopy), navigation or implanted devices. Reducing the amplitude of such noise will result in higher reproducibility with fewer trials. It has been proposed that to achieve this result, recordings be acquired from multiple derivations and only those be selectively utilized that have the highest signal-to-noise ratios rather than the utilization of recordings from standard diagnostic laboratory derivations [22]. Ultimately, even after averaging, what remains is an SEP estimate distorted by residual noise [22]. Although these responses will contain some noise, if the band-pass is well selected and the noise level is not too high, the responses will be quite reproducible. The number of trials per average may initially be set to begin at perhaps 300 trials but will depend on the signal-to-noise ratio and the urgency of reporting a result to the surgeon [22, 94, 106]. In some cases, such as during temporary occlusion of an intracranial vessel, the surgeon may wish to be informed quickly of changes in the evoked responses. In these cases, if signal quality is sufficient, adequate upper and/or lower extremity SEPs can sometimes be obtained with a few trials [22] e.g., 128 trials or less. If the number of trials is reduced, the person providing the monitoring needs to be sure that the responses obtained are indeed real and not artifact. In order to do so, that person needs to assess their accuracy by their reproducibility which is done by visual inspection and fastidious trending of accurately placed cursors.

## Electrophysiology

### SEP response origins

On a cellular level, SEPs are bioelectric events in neurons subsequent to changes in ionic conductance. Coincident cellular events combine to generate electric fields (potentials) sufficient to be detected during intraoperative monitoring of the nervous system. On a tissue or systems level, SEPs arise from two generator types. One type of generator results in volume-conducted perturbations in the body’s electric field and emerges from the physiology at several anatomic loci [107]. Most commonly, they originate from simultaneous bulk activation of synapses such as the thalamocortical afferents believed to contribute to the N20 peak following activation of a distal upper extremity nerve. Perturbations of the body’s electric field can also derive from action potentials in axonal components of the SEP pathway. Action potentials propagating in axons that pass through a change in the composition of the surrounding tissue are also generators of this type. Generally, whereas their amplitude decays with distance from their source, these generators result in stationary potentials. Thus, for a given activation site, their latency is the same at each recording site. Furthermore, under the right conditions and despite their often small amplitude, they can be observed with an electrode at a distance from their source, in which case they are referred to as far-field potentials. When observed using electrodes near their source, they are referred to as near field potentials [107, 108].

The other type of generator corresponds to action potentials conducted in nerve fibers, such as the waveform captured via recording electrodes over the nerves traversing the popliteal fossa (PF) subsequent to activation of a distal lower extremity site. These are propagated potentials (traveling waves). Therefore, the latency of the observed potential depends on the recording and stimulating inter-electrode distance. The potentials from this generator type require that the recording electrode be close to the source of the observed potential as they are not volume conducted well despite their often large amplitude.

Components of the SEP may originate from one or a combination of these physiologic generator types. For example, the multi-phasic waveform of the potential captured at the lower back after activation of a distal lower extremity nerve has a peak that is a stationary potential, corresponding to segmental synaptic events within the lower spinal cord, and another that is a propagated potential, corresponding to ascending action potentials in the afferent nerve fibers [104, 109].

Coincident activation of excitatory synapses of the cortical pyramidal cell results in an influx of positive ions, a so-called current sink. This influx is matched by a corresponding efflux of positive ions at a distant location creating a current source [107]. On a cellular level, these may occur respectively in the pyramidal neuron basal and apical dendritic arbors, or vice versa. When this occurs in multiple neurons simultaneously, such as upon artificial electrical activation of a distal nerve in an extremity for monitoring SEPs, distinct, large, extracellular regions of opposite polarity emerge and can be observed at a distance. The electrical fields generated in this way result in directional polarity resulting in an electrical dipole. The dipole orientation relative to the body’s surface and recording sites typically employed to capture SEPs are critical. Those oriented tangentially relative to the surface, such that the positive and negative end of the dipole is a similar distance from the surface (i.e., parallel to the surface), can be observable at a distance from their origin and the differing polarities on either side of the dipole can be distinguished. On the other hand, those that are oriented radially, such that the positive and negative end of the dipole are different distances from the surface (i.e., perpendicular to the surface), can only be observed locally, and the polarity of the more superficial portion of the dipole dominates the surface recorded potential. The polarity does not change on the surface​​ which is helpful for example when performing sensory mapping [49, 107, 108, 110, 111].

The nomenclature that is used to designate the peaks and valleys of SEP waveforms uses N and P, respectively, to designate the surface polarity of the recorded signal. The N potential is recorded on the surface negative side of the dipole, and P potential is recorded on the surface positive side of the dipole. When an N potential is acquired via an electrode connected to the negative (active/inverting) input of the differential amplifier, it is deflected upward on the screen. In contrast, when the P potential is acquired via an electrode connected to the negative (active/inverting) input of the differential amplifier it is deflected downward on the screen. If an N potential is recorded via the positive (reference/non-inverting) input of the differential amplifier, it will deflect downward on the screen. The output amplitude also depends upon the signal recorded by the reference electrode as compared to the active electrode and an integer is used to denote the nominal post-stimulus latency of the signal in normal adults. Illustrations of sample SEP waveforms with the requisite peaks and valleys marked using this nomenclature appears in a previously published guideline [13].

### Peripheral responses

Peripherally derived SEPs are generated in the nerve and the corresponding components of the respective plexus that subserve the portion of the sensory pathway activated by the distal stimulation. Peripheral SEPs are near-field propagated potentials observed as multiphasic waveforms that emerge from the current loops of the compound nerve action potential [107, 112].

For upper SEPs, peripheral potentials are traditionally captured with recording electrodes placed over the brachial plexus at ERB’s point (EP). Typically, the electrodes over the left and right Erb’s points are simply referred to each other, i.e., as EP_i_ to EP_c_ (where the subscript “i” and “c” denote ipsilateral and contralateral to the stimulated limb), since the electrode at EP_c_ is relatively inactive. Alternately, EP_i_ is referred to a frontal scalp electrode such as Fz.

For lower SEPs, peripheral potentials are traditionally captured in a bipolar fashion from recording electrodes placed one above the other behind the knee in the popliteal fossa (PF). Typically, the distal and proximal electrodes are simply referred to each other, i.e., as PF_d_ to PF_p_ (where the subscript “d” and “p” denote distal and proximal on the leg). However, a single PF electrode can also be paired with an electrode outside the PF, such as in the hamstring superior to it. For the waveform to have morphology as described below, with the negative peak deflecting upward, the active electrode should be PF_d_ in the PF_d_ to PF_p_ derivation.

Peripheral potentials corresponding to upper and lower SEPs are similar morphologically such that in the center of these multiphasic waveforms is a sharp, upward deflecting, negative peak that in normal, healthy adults occurs at approximately 9 ms. This is delineated as N9. Trailing the N9 is a downward-deflecting, positive trough. The absolute timing of the N9 peak reflects the waveform’s latency and the relative difference between the N9 peak and the trailing trough reflects the waveform’s amplitude.

Other recording derivations for peripheral upper extremity SEPs have been suggested, particularly because the EP_i_ to EP_c_ potentials suffer from poor reproducibility due to an unfavorable signal-to-noise ratio and can require significant averaging to resolve. These include derivations that capture Erb’s point potentials, such as referring EP_i_ to an electrode over the contralateral mastoid [94]. This has been demonstrated to substantially improve the signal-to-noise ratio of the Erb’s point potential, thus reducing the required trials per reproducible average. Some derivations capture responses at other peripheral locations. Bipolar recording from electrodes placed at the antecubital fossa results in a large peripheral response that is resolvable with little or no averaging [94]. While this waveform’s morphology is similar to that captured near EP, its latency is earlier due to the shorter interelectrode distance between the sites of stimulation and recording.

### Segmental and subcortical responses

SEPs generated within the CNS but below the cortical level derive from several locations in the pathway. The potentials captured depend on the location of the electrodes in the recording derivation. They can be grossly categorized by the presumed location of the generator of the potential as either segmental (or cervical for upper extremity SEPs and lumbar for lower extremity SEPs), originating from the spinal cord, or subcortical, originating from deep and/or low brain structures such as the brainstem and thalamus. The location of the electrodes used in derivations for capturing these potentials is dictated by access and the relative impact that the information collected by that derivation has on mitigating the neurologic risks of the specific surgery.

The derivations commonly comprise an electrode placed in the back or front of the neck or head. Traditional derivations pair these with a central, frontal electrode (e.g., Fz, Fpz) or a non-cephalic electrode (e.g., EP_c_). However, a mastoid reference has been shown to improve the signal-to-noise ratio [94]. Some practices reverse the arrangement of the recording electrodes in their derivations, preserving the polarity while flipping the direction of the deflections described in the following.

For upper extremity SEPs, using the posterior derivation of CS5 – EP_c_, a negative, upward deflecting far-field stationary segmental potential is observed that occurs roughly 13 ms after being elicited at the wrist. This is delineated as the N13 peak which may originate from multiple sources within the cervical spinal cord. Due to the horizontal orientation of its dipole, it will appear to have the opposite polarity when recorded with an anterior derivation [109, 113–115]. Using the scalp derivation of CP_i_ to a non-cephalic reference such as EP_c_, a nearly coincident positive, downward deflecting far-field propagated subcortical potential occurring at 14 ms is also often observed. This is delineated as P14, which is believed to originate from the proximal medial lemniscus pathway in the upper medulla [109, 113–120]. In this derivation, P14 is followed by a negative upward deflecting far-field stationary subcortical potential occurring at approximately 18 ms. This surface potential, delineated as N18, is believed to derive from the cuneate nucleus, rostral brainstem structures or within the thalamic nuclei [109]. The absolute timing of the P14 trough is the waveform’s latency, and the relative difference between the P14 trough and the P18 peak reflects the waveform’s amplitude.

A common practice is to employ CS5-Fpz to collect upper extremity SEPs, particularly since this derivation is useful for capturing lower extremity subcortical potentials and is likely already in the monitoring protocol. The waveform often has a characteristic “W” shape with an initial negative, upward deflection at approximately 13–14 ms. This is followed by a downward deflection. Multiple structures contribute to the waveform as captured using this derivation [121]. In this instance, the timing of the initial peak is the waveform’s latency, and the relative difference between the initial peak and the trailing trough reflects the waveform’s amplitude.

For lower extremity SEPs, the distinction between the generators for the segmental and subcortical potentials is clearer due to their physical separation. A segmental potential is captured from the lumbar spine and is often referred to as the lumbar potential (LP). Using a derivation including an active electrode over the T12 or L1 spinal level with a reference over the iliac crest contralateral to the stimulation (IC_c_), an upward deflecting, negative, near-field, stationary, segmental potential occurring at approximately 22 ms is observed. This is delineated as N22 and is reported to reflect the synaptic activity of intrinsic circuits of the lower spinal cord [109]. N22 is followed by a shallow trailing trough. The absolute timing of the N22 and the relative difference between the N22 peak and the trailing trough reflects the waveform’s latency and amplitude, respectively. In some instances, the waveform has multiple peaks, one of which is the N22 or its equivalent and one of which is a mixed nerve propagated potential coursing through the spinal roots, the latency of which is determined by the location of the recording electrode. This additional wave is not typically tracked during monitoring.

Subcortical potentials corresponding to lower extremity stimulation can be captured with an Fpz electrode referred to an electrode at CS5 [17]. The resulting waveform is biphasic with a small initial positive, downward deflecting, far-field subcortical potential, occurring at 31 ms, delineated as P31. This P31 is believed to be derived from the dorsal column nuclei and/or the caudal medial lemniscus [109]. It is considered the equivalent of the P14 following stimulation of the upper extremity. P31 is followed by a larger negative, upward deflecting, far-field stationary subcortical potential occurring at approximately 34 ms. This potential, delineated as N34, is considered the equivalent of the N18 following stimulation of the upper extremity and is believed to derive from multiple sources, including the brainstem and synaptic activity within the thalamic nuclei [109].

### Cortical responses

The cortical SEP is generated from synchronous thalamocortical synaptic activity at locations predominantly within the postcentral gyrus. These potentials are detectable over much of the scalp, even at a distance from the generator. Upper SEPs are maximal when captured by a derivation including an active electrode located over the lateral, postcentral gyrus on the side of the head opposite the stimulated upper limb consistent with the lateral representation of the upper extremity in the S1. Lower SEPs are maximal when captured by a derivation including an active electrode in the midline consistent with the representation of the lower extremity in the S1, which is tucked in the medial, interhemispheric bank. Lower SEPs are also maximal ipsilateral to the stimulated limb due to the phenomenon of “paradoxical lateralization.” For upper and lower SEPs, the reference electrode in these derivations can be non-cephalic but is typically another site on the scalp. Lateralized recordings should be paralleled by recordings from the opposite side of the scalp. This allows for troubleshooting inadvertent erroneous electrode misplacement, such as left–right switching of the scalp and/or stimulator channels. This also provides an alert for a patient with a non-decussating pathway [122].

Traditional scalp derivations for capturing upper extremity cortical SEPs include CP_c_–Fpz or Fz, or CP_c_–CP_i_. Recording with these derivations, a set of near-field stationary potentials are observed as a characteristic biphasic waveform with an initial negative, upward deflection that occurs roughly 20 ms after being elicited at the wrist. This peak is delineated as N20. The N20 is often followed by a downward deflecting, positive potential at approximately 30 ms. This trough is delineated as P30. The N20 peak and P30 trough emerge from tangential dipoles in the anterior bank of the postcentral gyrus. There may also be an intervening positive, upward deflection that occurs at 25 ms, delineated as P25. This peak can only be observed when the recording electrode is directly over the generator, as the potential derives from a radial dipole at the vertex of the anterior bank of the postcentral gyrus [79, 123]. The P25 potential impacts the appearance of the waveform, altering it from bi- to tri- phasic. It is the absolute timing of the N20 that is the waveform’s latency and the relative difference between the N20 peak and the P30 trough that reflects the waveform’s amplitude [124–126]. The trough appearing before (to the left of) the P25 peak may also serve as the lower boundary of the amplitude should a measurable P30 be absent, irregular, or inconsistent. The pre-P25 downward deflection might also be chosen as the trough if the P30 amplitude is smaller.

Lower extremity SEPs have been captured using recording derivations such as CPz-Fpz or Fz and CP_i_-CP_c_. Using these derivations, a biphasic waveform composed of near-field stationary potentials is observed. This waveform consists of an initial downward deflecting positive potential followed by an upward deflecting negative potential that occur roughly 37 ms and 45 ms, respectively, after stimulation at the ankle. These are, therefore, delineated as P37 and N45. The absolute timing of the P37 reflects the waveform’s latency and the relative difference between the P37 trough and the N45 peak reflects the waveform’s amplitude. Note that the latency of the P37 waveform may differ between these recording derivations [127]**.**

Other derivations have also been recommended, particularly those that result in recordings with favorable signal-to-noise ratio, such as CP_c_–CPz and its inverse for upper and lower SEPs, respectively [22, 94, 106]. These derivations have varying levels of acceptance and implementation. The derivation(s) chosen should be those that result in potentials with the best signal-to-noise ratio and the least trials per average as part of an overall signal optimization plan.

In some instances, such as to avoid the exposure site of a craniotomy, scalp recording electrodes are displaced. Whether the intent is to avoid the sterile field or to incorporate recording derivations with optimal signal-to-noise ratio, electrodes placed away from the standard locations often result in waveform morphological characteristics that differ from those captured by traditional derivations as described above. In some instances, this may be a difference in the amplitude and/or sharpness of the obligate peaks but may also include inversion of the peaks and troughs, particularly for recording locations anterior to the central sulcus [94, 106].

### SEPs in neonates and children

Cortical, subcortical, and peripheral SEPs recordings have been reported in premature and term infants and children [128–131]. The central and peripheral neurons mature synchronously, with the peripheral maturing early [132]. Thus, the conduction velocity of the central and peripheral nervous systems is slower in infants [129, 133]. There are significant differences in the central sensory conduction time values between the SEP parameters in children younger than 12 months and 1 to 12 and 12 to 17-year-old children. The age-related reduction in the sensory central conduction time and the increased amplitude of the cortical responses may reflect the myelination of somatosensory pathways and improved nervous system integration. Maturational factors indicate myelination occurring within the thalamus from 34 weeks gestation onwards [133]. Cortical SEPs may be difficult to record in healthy infants at birth and up to as old as three months. Upper extremity responses are likely to be present earlier than lower extremity responses. When present, the SEP component latencies are shorter in infants and children primarily due to size, and with growth and maturation, these latencies will increase. These changes are mainly a reflection of the elongation of the peripheral nerves and the central somatosensory pathways. However, as these elongation processes occur, they are partly counterbalanced by the pathways becoming myelinated and nerve fiber diameters increasing, resulting in faster conduction velocities. In addition, maturation of synaptic transmission is also occurring. These events simultaneously happen until children reach 6–8 years of age when central times are comparable to an adult. At that time, any further latency changes result from changes in stature [134]. It has been reported that waveforms can be observed in premature infants and full-term newborns. The authors concluded that myelination is determined by conceptional age, and is unrelated to the gestational age at birth [135].

Premature and young infants represent unique challenges when interpreting SEP data due to the immaturity of their sensory pathways and cortex. An essential SEP component may be absent not because of a pathological process but because of a maturational standpoint. There is high variability when interpreting SEP data across studies. Prematurity itself, in the absence of perinatal brain injury or other complications, does not seem to be responsible for alterations of the central somatosensory system in at-term corrected age newborns compared with full-term neonates [136].

## Anesthesia and physiological considerations

### Anesthesia considerations

Anesthesia can impact evoked potential responses, and clear communication between the IONM and anesthesia teams is essential to aid the anesthesiologist in planning a maintenance anesthetic regimen that will incorporate IONM needs while he/she also considers patient comorbidity concerns and other surgical requirements. Evoked potentials that depend on polysynaptic function, such as cortical SEP responses, are most impacted by anesthetic agents, while subcortical and peripheral SEP responses are less sensitive [103, 137]. This is because anesthetic agents are understood to exert their mechanism of action(s) via interaction at specific ion channels that alter synaptic transmission and membrane potentials, with each agent differentially targeting a variety of ion channels in certain areas of the brain and spinal cord [138]. Anesthetic effects on cortical SEPs are generally dose-related and tend to correspond with anesthetic effects on the electroencephalograph (EEG), since both depend on cortical synaptic transmission activity [139]. There is usually a larger negative impact on evoked potential amplitude than on latency, because the effect of anesthetics tends to be greater on synaptic transmission than on axonal conduction [103]. In routine clinical practice, several anesthetic agents are frequently incorporated into one anesthesia maintenance plan for a “balanced” regimen that aims to synergistically maximize the goals of anesthesia while minimizing undesired side effects. When planning the maintenance anesthetic regimen, the effect of each anesthetic agent on the specific IONM modalities being employed must be considered.

#### Inhalational anesthetic agents

The most commonly used anesthetic agents to be included in anesthesia maintenance regimens are the halogenated volatile inhalational agents (e.g., sevoflurane, desflurane, and isoflurane) [138]. These modern inhalational volatile anesthetic agents seem to have similar effects on SEPs at steady state concentrations [140]. At clinically relevant dosing, these agents produce a modest dose-related reduction in amplitude and increase in latency of cortically recorded SEP responses [141]. Thus, in neurologically intact patients, the concentration of these inhaled volatile agents is usually limited to 0.5 to 1 minimal alveolar concentration (MAC) for cortical SEP monitoring [140]. Desflurane followed by sevoflurane are less lipid soluble than isoflurane and therefore allow faster transition to a total intravenous anesthesia (TIVA) technique when baseline signals are unacceptable in the presence of volatile anesthetic. Nitrous oxide differs from the halogenated inhalational agents, and, at equipotent concentrations, it depresses evoked potentials more than halogenated agents [137]. When combined with other inhalational agents, nitrous oxide has a synergistic depressant effect on cortical SEPs [142]. As such, nitrous oxide is usually avoided while monitoring cortical SEPs [140]. SEPs recorded from the brainstem, spinal cord, and periphery are either only minimally or not impacted by volatile anesthetics [140].

#### Intravenous analgesic agents

A variety of intravenous anesthetic agents are available. Their effect on evoked potential responses depends on the specific receptors and pathways targeted by the agents [137]. In general, though, intravenous anesthetics have a less depressant effect on evoked potentials as compared to inhalational anesthetics [140]. The most common intravenous anesthetic agent for maintenance of anesthesia is propofol. A TIVA technique with propofol facilitates evoked potential monitoring, as the changes in evoked potential amplitude with propofol are smaller than with equipotent doses of halogenated agents. Propofol-based TIVA also aids in obtaining reliable MEPs when multimodal IONM is utilized [143] [144]. Although propofol is less suppressive than inhaled volatile anesthetics under steady-state conditions for evoked potential amplitudes, propofol also attenuates evoked potential amplitudes in a dose-dependent manner [140]. Fortunately, adjusting anesthetic depth and the impact on evoked potentials is fairly simple with propofol due to its relatively rapid metabolism and redistribution [139]. During critical portions of surgery, it is important to maintain stable propofol blood levels so as to not confound evoked potential monitoring [145]. For example, during significant intraoperative blood loss, the serum concentration of propofol will increase (because of altered pharmacokinetics and decreased volume of distribution), so the propofol infusion rate should be titrated down to minimize its potential negative impact on evoked potentials while also maintaining a focus on restoring intravascular volume and hemoglobin level [145].

Barbiturates have a similar effect on evoked potentials to that of propofol [140]. However, fewer barbiturates are clinically available than in the past, and their use is complicated by a longer half-life than propofol. This makes titration during TIVA that allows for prompt emergence and neurological examination challenging [140]. One ultra-short acting barbiturate, methohexital, is occasionally currently used intraoperatively, however [146]. Its use has been reported in a small series to lead to acceptable cortical SEP monitoring [147].

Benzodiazepines are another class of intravenous hypnotics relied on less intraoperatively today than in the past. Typically, the benzodiazepine midazolam may be administered in a small dose as a sedative and amnestic agent before induction of anesthesia. However, for cases involving general anesthesia and IONM, as well as when a prompt emergence and neurologic exam are desired, additional administration of midazolam is unusual [148]. Nonetheless, it is recognized that midazolam, at higher doses consistent with induction of anesthesia requirements, produces depression of cortical SEP amplitude and has minimal effects on cortical SEP latency and on subcortical and peripheral sensory evoked responses when administered as the sole agent [149]. Recent introduction of a new short-acting benzodiazepine, remimazolam [148], may require future evaluation as to its impact on SEPs and other IONM modalities [150].

Another intravenous anesthetic, etomidate, enhances cortical SEP baseline amplitudes without an effect on subcortical or peripheral SEPs [137]. The amplitude enhancement has been shown to occur within minutes after an etomidate intravenous bolus [151] and the amplitude enhancement has been attributed to heightened cortical excitability elicited by etomidate [137]. Constant infusion has been used to enhance SEP cortical recordings that were otherwise unsuitable for monitoring purposes [152] although clinical use of etomidate is limited by concern of adrenal suppression and postoperative nausea and vomiting [140].

Traditionally, TIVA techniques have included an opioid analgesic with a hypnotic anesthetic (usually propofol). In recent years, there has been increasing interest in opioid-sparing multimodal analgesia in anesthesiology, including for surgeries that use IONM [153]. It is thus necessary to consider the impact of these multimodal analgesia adjunct agents on IONM modalities, although currently available evidence does not allow definitive recommendations for specific multimodal analgesic regimens during IONM [154]. Nonopioid analgesics may have a primary benefit on cortical SEPs by decreasing the pharmacological requirement for sedative-hypnotic agents (e.g. propofol). Thus, careful titration of agents is essential for adequate IONM [154].

The intravenous anesthetic ketamine is one of the most common agents incorporated into multimodal analgesia regimens, and it has traditionally been regarded as having an augmentative effect on cortical evoked potentials [154, 155]. Although a depressive effect on MEPs with higher bolus doses has recently been reported, this was not found to be the case for SEPs [156].

Dexmedetomidine, another intravenous anesthetic agent, seems to have minimal to no effect on SEP monitoring, at least at lower doses [157–161]. Higher bolus doses may decrease amplitude and increase latency of cortical SEPs [162]. Perhaps the greatest benefit when dexmedetomidine is incorporated into a TIVA regimen with propofol is derived from a reduction in the amount of propofol required to achieve the desired depth of anesthesia [158]. Even when the infusion is held at a constant dose, dexmedetomidine plasma concentration will increase during the intraoperative course. During long surgeries, it is necessary, therefore, to decrease the infusion rate as the case continues to avoid a potentially deleterious impact on evoked potentials [154, 163].

Another currently popular multimodal analgesic is the local anesthetic lidocaine. Lidocaine does not seem to impact evoked potentials with routinely used infusion ranges [154, 164, 165]. A recent randomized crossover study evaluating the effect on evoked potentials of adding a lidocaine infusion to the anesthetic regimen did not find a significant difference in SEP amplitude with lidocaine incorporation [166].

Methadone is an effective analgesic for spine and other surgeries, and intravenous bolus dosing of methadone seems compatible with IONM [154, 167]. Methadone is unique as a multimodal agent in that it has both µ-opioid agonist and N-methyl-D-aspartate (NMDA) receptor antagonist activity, and it has a much longer elimination half-time than other opioids [168]. A recent prospective non-randomized study evaluated the effect of a routine clinical bolus dose of methadone on evoked potentials for up to 15 min post-bolus, and found a statistically (but not clinically) significant decrease in amplitude and increase in latency of SEPs [169].

#### Opioids

Opioids generally decrease the amplitude and increase the latency of cortical SEPs, but even relatively high-dose infusions still usually allow adequate monitoring [103, 137]. Bolus dosing will produce greater negative impact on cortical SEPs in a dose-dependent manner [169, 170]. Indeed, an adequate opioid dose, usually administered as an infusion, can be an essential component of the maintenance anesthetic regimen to help provide immobility when neuromuscular blockade cannot be used, as is often the case with multimodality IONM incorporating MEPs and/or EMG along with SEPs [171]. At very high infusion doses, remifentanil, a short-acting and commonly used opioid during cases involving IONM, was found to cause a 20 to 80% decrease in cortical SEP amplitude and a less than 10% increase in latency during spine surgery [172]. This finding likely emphasizes the need to titrate reduction in other anesthetic agents when using high-dose opioids or other adjunctive agents [140].

#### Muscle relaxants

Neuromuscular blocking agents improve the monitoring of SEPs by eliminating spontaneous background electromyographic noise [103, 140]. Also, observation of excessive myogenic artifact by the IONM team can be helpful information for the anesthesia team, as it may indicate the patient is “light” and suggests possible need for more anesthetic and/or analgesic [16].

#### Selection of anesthetic maintenance regimens

Some patient factors and comorbidities can make it more difficult to obtain adequate baseline evoked potential recordings. SEPs are expected to have a smaller amplitude and a longer latency in elderly patients [173]. Also, increased height and weight, lower extremity edema, neurologic deficit on exam, and history of neurologic disease have all been found to make it more difficult to obtain baseline SEPs [174]. However, with a facilitating anesthetic technique, evoked potentials can often be reliably obtained [175]. Recently, with modern monitoring equipment, standardization of recording techniques, and a facilitating anesthetic regimen (propofol and remifentanil-based TIVA technique or a regimen supplemented with less than 0.5 MAC of halogenated anesthetic agent), a series of consecutive cranial and spine surgeries reported a success rate for obtaining acceptable baselines of 98.1% for upper extremity SEPs and 90.1% for lower extremity SEPs [176].

The optimal anesthetic regimen for surgery involving IONM is controversial. Perhaps the best plan involves obtaining a stable anesthetic environment and then not varying the anesthetic regimen once adequate baseline signals are obtained [177]. Although straight-forward in theory, changing degrees of surgical stimulation at different phases of surgery often do require changing anesthetic depth intraoperatively, and communication between anesthesia and IONM teams regarding change in anesthetic technique or bolus administration is necessary [177]. It is important that significant changes in anesthetic technique not be made during critical surgical maneuvers.

Cortical SEPs can be obtained in most patients when a volatile anesthetic is limited to 0.5 MAC and supplemented with more evoked potential-facilitating intravenous medications. This usually consists of propofol as an additional hypnotic and an opioid for additional analgesia [178]. If inhaled volatile anesthetic agents are initially included in the anesthetic, it is critical that communication occur between the anesthesia and IONM teams in order to assess adequacy and reproducibility of baseline evoked potentials, and to convert promptly to a TIVA regimen if evoked potentials are not robust enough for high-fidelity monitoring. When an anesthetic technique is modified, adequate time (approximately 30 min) may be necessary to allow for recovery of signals [177]. In patients with preexisting neurological disease or deficits, avoiding inhaled volatile anesthetic agents might be required to elicit an adequate response [140, 178]. We recommend starting with a more evoked potential-facilitating TIVA technique in this situation [175].

### Physiological considerations

In addition to providing anesthesia, a core role of the anesthesiologist is to maintain patients’ physiologic homeostasis. This is also critical for maintaining stable IONM signals. As such, close communication amongst the surgical, anesthesia, and IONM teams is again essential to understand changing physiological parameters and the impact these may have on evoked potentials [140, 177].

#### Temperature

Temperatures in the OR are generally well below body temperature. As a result, it is not unusual for a patient’s temperature to drop during surgery. The temperature of the room, the length of the surgery, and the amount of surgical exposure will all contribute to the patient’s heat loss and resulting body temperature. Diminished body temperature will affect the metabolism of the drugs used for anesthesia. To counteract this effect, anesthesia personnel often use forced air warmer blankets to maintain the patient’s body temperature. Another effect of diminished body temperature is a decrease in neural conduction velocity with a resulting increase in SEP peak latencies [179, 180]. SEP changes with minor variations in temperature are gradual (roughly 0.75–1.0 ms increase in latency of the N20 for every 1 °C decrease in nasopharyngeal temperature) and occur without significant amplitude changes [98]. Mild hypothermia (32 °C), perhaps counterintuitively, increases amplitude of SEPs in rats and humans [181]. This likely occurs due to a hyperexcitable cortex and reduced neurotransmitter catabolism [181, 182]. However, with very low temperatures, the cortical evoked responses disappear (roughly 22 °C) [180] and subcortical, spinal, and peripheral SEP responses with elevated peak latencies may be relied upon for the monitoring of somatosensory function. Though subcortical responses have been reported to disappear between 13 and 16 °C [183, 184], it is clear this occurs at a thermal point below which the cortical response is lost.

#### Blood pressure

Blood pressure affects the perfusion of neural tissue. A certain amount of neural perfusion is necessary to meet the metabolic demands of the tissue. If these demands are not met, the electrical activity of the tissue will begin to shut down. Although cortical blood flow is not often measured directly intraoperatively, it has been reported that cortical SEPs begin to change when cortical blood flow drops below 18 ml/100 g/min [185–187]. The amplitudes drop and the response latencies systematically lengthen. Further ischemia causes approximately a 50% decrease of cortical SEPs when the cortical blood flow drops below approximately 15 ml/100 g/min as an early warning sign [185–187]. The degree and duration of low flow below this warning threshold appear to correlate with the degree of permanent neurological damage [185–187]. Additional drops in blood flow to the brain, particularly if they are sustained, will result in cellular damage and irreversible changes in electrical activity [185–187].

In general, cortical evoked potentials appear to be minimally attenuated when systolic blood pressure is kept stable at 80 mmHg [98, 179]. However, the degree of degradation of cortical SEPs with decreases in blood pressure varies between individuals. Pressures which produce no SEP changes in one patient may produce significant changes in another. Cortical SEP changes which cannot be otherwise explained may result from hypotension and simply raising the mean arterial pressure can result in restoring SEP response losses [188]. Because of autoregulation, the critical threshold at which ischemic changes in the SEP responses occurs is dependent upon the patient’s “normal” outpatient blood pressure. It is also dependent upon the presence of cerebrovascular disease. Intracranial pressure (ICP) has also been shown to have an effect on SEPs during IONM. Because of pressure-related effects on cortical structures, reduced amplitudes and increased latencies have been observed when ICP is increased [137, 189].

Subcortical and spinal SEP recordings are more resistant to ischemia than cortical SEP recordings due to the larger proportion of white matter than grey matter that comprise the pathway at these levels. Therefore, these signals may continue to demonstrate measurable electrical signals even after blood flow to the generator sites has ceased for several minutes.

In the spinal cord, it is important to understand how one measures spinal cord perfusion pressure (SCPP). Although it cannot be normally monitored in the OR, SCPP equals mean arterial pressure (MAP) minus intraspinal pressure (ISP or intrathecal cerebrospinal fluid pressure [190]. During certain surgeries, if IONM changes raise concerns for spinal cord ischemia, it may prompt the placement of a lumbar drain to remove cerebrospinal fluid so as to improve spinal cord perfusion and raise the MAP [141].

#### Metabolic factors

Oxygen supply is necessary to meet the metabolic demand of the neural tissue mediating the SEP response. Cortical SEPs are the most sensitive in this regard. Mild acute hypoxia however, does not affect the SEP in humans [191]**.**

SEPs are resistant to transiently reduced levels of carbon dioxide (i.e., hypocarbia or hypocapnia) with subcortical structures generally demonstrating greater resistance than cortical structures. In anesthetized patients, mild, acute, hypocapnia has no effect on median nerve cortical SEP amplitude but moderately decreases their latency [192]. Similarly, hypocapnia results in minor decreases in latency and increases in amplitude of tibial SEPs in awake volunteers [191]. This decrease in SEP latency associated with decreased CO2 is attributed to increased conduction velocity. In contrast, it has been reported that tibial nerve SEPs were not changed is with hypocapnia. Hypercapnia on the other hand does not appear to affect the SEP amplitude or latency in either anesthetized patients or awake volunteers [191, 193].

While it is likely rare to have acute intraoperative SEP changes due to electrolyte abnormalities, some electrolyte changes can impact SEPs. For example, a hypocalcemia group post-parathyroidectomy demonstrated increased median nerve SEP amplitude and longer recovery functions upon multi-pulse challenge of the SEP [194]. The authors suggested that this is indicative of an abnormality of fundamental synaptic function. Potassium (K^+^) abnormalities could also plausibly impact SEPs, since it is easy to envision a direct relationship between K^+^ levels and SEP characteristics owing to the critical role K^+^ ions play in establishing the resting membrane potential and in repolarization following an action potential. However, we are not aware of direct studies evaluating the impact of hypokalemia or hyperkalemia on SEPs.

## Applications/indications

### SEP monitoring

#### Spinal cord monitoring

Orthopedic spine surgery is the oldest and most common indication for SEP monitoring even though motor deficits resulting from these surgeries are the main concern [50]. The original rationale for SEP usage during these procedures was based on the proximity of the motor and sensory pathways. It was theorized that if a cord compromise were to occur, it would involve both pathways resulting in SEP changes and intervention. As a result, SEPs have been widely used to assess spinal cord function [2–4, 31, 40, 61, 62, 78, 195–232]. Although SEP monitoring alone halves the risk of motor injury, motor deficits due to small lesions may still occur without any SEP changes and the opposite may occur as well [51, 202, 233–237]. Now that MEPs are widely available, SEPs are still useful during spine surgery as a complimentary monitoring modality. For example, SEPs offer the benefit of continuous acquisition and monitoring, which is not the case for MEPs. Also, when MEPs are unobtainable, (e.g. due to significant motor weakness), SEPs can offer some monitoring capability. Prospective studies validating the efficacy of multimodality IONM are lacking but there is a growing body of evidence supporting its use during spinal surgery including surgeries for complex deformities and spinal cord tumor resections [238].

##### Cervical spinal cord monitoring

SEPs are widely used to assess cervical spinal cord function [200, 204, 206, 210, 218, 220, 222, 229, 239–242]. In order to accurately do so, the elicited responses must be completely rather than partially conducted through the surgical site or sites at risk. Therefore, care must be taken when selecting stimulation sites. Peripheral nerve responses are mediated by more than one spinal nerve root as they enter the spinal cord. The responses elicited by median nerve stimulation are mediated by several nerve roots (C5-T1). Although these responses are easy to elicit and are normally quite large in amplitude, they may not be an effective monitoring tool if they are mediated by nerve root components that are located above the surgical site. If the surgical site is above and includes the level of C7, ulnar nerve responses may be more effective monitoring tools in this case [76]. Monitoring cervical spinal cord function also utilizes lower extremity SEPs. The choice of stimulation sites for eliciting these responses is generally the tibial nerve at the ankle and other sites as needed.

Several recording sites can be used to monitor upper extremity SEP activity. The recording site over the cervical spine may be the most important SEP recording site because the responses are usually from a location(s) above the sites at risk and are generally unaffected by the anesthetic drugs used for patient management. These responses have several peaks because of multiple generator sites (see Sect. 5.3). The N13 peak has multiple generators some of which are below the medulla and some at the cervico-medullary junction. The P14 is generated above the level of the spine. Therefore, the appropriate montage must be utilized to clearly distinguish N13 and P14 peaks. It must also be remembered that SEPs assess sensory function mediated only by the dorsal column pathways and not motor function. Therefore, surgical insults to the anterior spinal cord or blood supply to the anterior spinal cord may not be detected by SEPs. There have been several reports of false-positives and false –negatives associated with the use of this technique [233–238]. As a result, safety concerns have been raised as to whether SEPs can be used as a standalone monitoring technique [238]. Although the evidence appears to support increased detection of neurological injuries in cervical procedures using SEPs in conjunction with MEPs [240, 241], controversy remains within the spine community as to the utility of SEP monitoring during cervical spine surgery [239, 243] and whether monitoring is needed for routine non-complex cervical spine procedures [244–247].

Surgical examples include the following: anterior and posterior cervical spinal fusions [204, 210, 248–251], spinal cord lesions [41], dorsal column mapping [38–40, 244], spinal cord stimulation lead placement [42, 43].

##### Thoraco-lumbosacral and cauda equina monitoring

Neurological injury is a much-dreaded complication in spine surgery and although its occurrence is relatively infrequent, it has the potential to result in serious postoperative motor and sensory deficits. Since the introduction of SEP monitoring in the 1970s, largely in the setting of spinal deformity correction, the rate of neurological injuries in scoliosis surgery has been significantly reduced [213]. In an effort to avert neurological complications for all types of spinal surgery, an increase in the utilization of IONM has occurred in recent years. However, because false-negative SEP changes have been reported in several studies, the use of SEPs as a singular tool for spinal cord neuromonitoring has largely been abandoned in favor of multimodality monitoring which often includes multi-extremity SEP, MEP and EMG monitoring. A panel of experts reviewed the results of a comprehensive literature search and identified published studies relevant to the clinical question of whether IONM predicts surgical outcomes. These experts concluded that IONM utilizing SEPs in conjunction with transcranial MEPs “is established as effective to predict an increased risk of the adverse outcomes of paraparesis, paraplegia, and quadriplegia in spinal surgery” [19]. Numerous professional societies have endorsed this study and its conclusion.

In lumbosacral procedures, nerve root rather than spinal cord function is of paramount importance because only the thecal sac and nerve roots are at risk below the conus medullaris (L1–L2) [225]. In the lumbar spine, SEPs have been shown to have a sensitivity of 29% and a specificity of 95% [252]. Current consensus favors the use of SEPs, MEPs, and combined spontaneous and triggered EMG during lumbosacral interventions [225, 227, 253, 254]**.**

Upper extremity SEPs, although insensitive to changes in thoraco-lumbosacral spine function, can be useful for detecting functional changes associated with arm positioning during thoraco-lumbosacral surgical procedures. In addition, when changes in lower extremity SEPs occur, the status of upper extremity SEPs can be helpful for interpretation, such as for identifying possible global effect causes from anesthetic medications.

Surgical examples include the following: spinal deformity correction and repair [78, 197, 203, 213, 221, 223, 224, 246, 255, 256], dorsal column mapping [38–40, 244], spinal cord stimulation lead placement [42, 43], degenerative thoracic and lumbar fusions [196, 202, 257–260], interventional procedures [261], abdominal aortic aneurysm repair (AAA) [207, 212, 226, 230, 231, 262], removal of spinal cord tumors [263], and spinal fracture repair [214]. However, the use of IONM for degenerative lumbar surgery, and in particular procedures not involving instrumentation, remains controversial and should be considered on a case-by-case basis [260].

#### Peripheral nerve and plexus monitoring

SEPs can be used to assess the functional status of peripheral nerves and plexuses [23–29]. They are also useful for identification purposes and for assessing functional continuity. These anatomical structures consist of both sensory and motor nerve fibers. The responses recorded directly from these structures as a result of distal peripheral nerve stimulation are compound nerve action potentials (CNAPs) and consist of mixed orthodromic and antidromic sensory and motor activity [264]. It is not until the ascending responses are recorded from more proximal sites over the spinal cord or higher that they represent true somatosensory (SEP) responses.

Even when nerves are not surgically exposed, their function can still be placed at risk. This can be the result of a surgical maneuver or of positioning [67, 70, 71, 265–271]. Peripheral stimulation can be used to elicit SEP responses from these nerves and the resulting responses are typically recorded from the scalp or over the spine; sites proximal to where their function has been placed at risk [272]. The use of pudendal nerve evoked potentials provides a means of monitoring lower sacral nerve root function that conventional SEPs provide for higher spinal levels [273]. Its use is also important in monitoring of cauda equina and conus tumor surgeries [30].

Surgical examples include the following: peripheral nerve repair [274], position-related ulnar nerve and brachial plexus dysfunction [275–277], avoidance of neuropraxia during shoulder arthroscopy [25], protection of sciatic nerve function during total hip arthroplasty [24, 26, 278, 279], acetabular surgeries [104], pudendal nerve monitoring for surgical fixation below the S1 level [273], cauda equina and conus tumor surgeries [30, 272], peroneal nerve stimulation at the top of the foot for protection of the fibular head [82] and saphenous nerve stimulation during lumbar spine surgery [85].

#### Nerve root monitoring

Nerve root function can be assessed using monitoring techniques of sensory and/or motor function [11, 20, 21, 273, 280–284] that do not include mixed nerve stimulation. The SEPs that are elicited by mixed nerve stimulation are mediated by several cervical or lumbo-sacral nerve roots [11] as they enter and ascend the spinal cord. These responses may appear normal despite the presence of a nerve root whose function is abnormal [11]. This is thought to result from the abnormal function being masked by the responses mediated by other nerve roots whose function is normal [11]. Therefore, in order to test the function of individual nerve roots, body areas innervated by a single nerve root (known as dermatomes) can be electrically stimulated. The responses that result from this form of stimulation are called DSEPs. DSEPs have been used to intraoperatively assess nerve root function. They are sensitive to nerve root compression and mechanical manipulation [11]. However, it is questionable as to whether they are sensitive to nerve root decompression [11]. They can detect a misplaced pedicle screw but only when the screw contacts and mechanically irritates a nerve root. As a result, they are ineffective when no contact occurs [11]. In addition, DSEPs are an averaged response and require at least a few minutes to detect and confirm a mechanical insult. Whether DSEPs are an adequate intraoperative monitoring modality for detecting nerve root injury is still controversial [284]. The major shortcomings of the DSEP technique have been addressed by the use of motor pathway assessment techniques. These techniques are discussed in the ASNM MEP and EMG position statements [285, 286]. As a result, the intraoperative use of DSEP responses is now rarely if ever utilized.

Surgical examples where SEPs are used to assess nerve root function include: cauda equina and conus tumor removal [30], and the release of tethered cord [287–290]. However, a drawback of SEP monitoring during these procedures is that there can be an overlap of an adjacent root that can mask a single nerve root injury as discussed above [291]. Surgical application examples of DSEPs have included the placement of pedicle screw instrumentation [11], and during surgeries for various degenerative spine disorders [273, 282–284].

#### Brain monitoring

During various surgical procedures when brain function is at risk, it is common to monitor these procedures using SEPs alone or in conjunction with other IONM modalities, including EEG and MEPs [35, 36, 44, 45, 292–312]. Loss of function can result from direct surgical insult or indirectly from tissue ischemia. Cases where tissue ischemia is of concern include craniotomies for aneurysm clipping or arteriovenous malformation and neck dissection for carotid endarterectomies [313–315]. The location of an aneurysm will generally define what areas of the brain are at risk for an ischemic event and what SEPs may be helpful for monitoring purposes. For instance, the middle cerebral artery (MCA) provides blood to the sensory area for the hand whereas the anterior cerebral artery (ACA) provides the blood supply to the sensory area for the leg. Clipping of an MCA aneurysm could result in a misplaced clip and compromised blood flow within the MCA or within lenticulostriate perforating vessels from the MCA that supply the thalamus and the white matter. As a result, the misplaced clip could result in a loss of the contralateral upper extremity SEPs but could also result in a loss of the lower extremity SEPs if blood flow in the perforating vessels is compromised. On the other hand, when clipping an ACA aneurysm, a misplaced clip may result in a significant change in the contralateral lower extremity SEPs with no change in the upper extremity SEPs. Such changes may or may not occur in conjunction with similar EEG changes. Carotid occlusion may affect both upper and lower extremity SEPs. However, it should be pointed out that there are limitations to the use of SEPs for vascular procedures. Their use is only sensitive to ischemic events which affect the SEP generator sites. SEPs may therefore be insensitive to ischemic events in other areas of the brain which do not receive their vascular supply from branches of the above-mentioned arteries. Thus, multimodal IONM, including SEPs, MEPs and EEG, seems optimal for many cerebral vascular surgeries [307, 316, 317].

Surgical examples include the following: Craniotomy for tumor removal [311] aneurysm repair [35, 300, 309], craniotomy for vascular surgeries [294, 297, 299, 306, 313, 315, 318], interventional procedures [111, 301] and carotid endarterectomy [36, 303–305, 308, 310].

### SEP mapping

#### Spinal cord mapping

SEPs can be employed to map the spinal cord dorsal columns. SEP mapping methods guide the localization of the dorsal median sulcus in surgery for intramedullary lesions when the surgical trajectory is via a dorsal myelotomy [40, 244, 319]. SEP-dependent methods for localizing the dorsal median sulcus can be categorized by the site of stimulation—those in which the dorsal columns are stimulated directly and those in which nerves in the periphery are stimulated [320]. The dorsal columns are directly activated at a low current intensity via a handheld stimulus probe or a specialized multi-contact spinal micro-electrode. Averaged SEP waveforms are obtained from scalp recordings using the CPi–CPc derivation corresponding to different stimulated locations across the horizontal axis of the spinal cord. The waveforms demonstrate opposite polarity when the left versus the right fasciculus gracilis is activated. That is, they will phase reverse across the dorsal median sulcus [37, 38, 321]. They often will also show a flattening when the stimulus is applied directly at the midline. Antidromic propagated potentials captured over peripheral sites elicited by this stimulation method may be included to simultaneously complement the map obtained via phase reversed cortical potentials [319, 322]. Alternatively, the dorsal median sulcus can be localized by evaluating the gradient of the amplitude and complexity of propagated potentials captured directly from the spinal cord dorsal surface following stimulation of distal peripheral nerves [39–41, 323]**.** Direct stimulation of and recording from the spinal cord must be performed by a neurophysiologist experienced in spinal cord mapping.

SEPs are also helpful in optimizing the location of spinal cord neuromodulation devices such as spinal cord stimulators [42, 43, 324]. In this instance, the SEP collision method, relies on the conduction block that occurs at the site of collision of action potentials traveling in opposite directions in the same nerve/tract [42, 43]. Spinal cord stimulator treatment includes a multi-contact electrode, called a paddle, placed surgically on the dorsal surface of the spinal cord. Using an external pulse generator, the spinal cord is stimulated through select locations on the paddle in order to focally produce antidromically propagated dorsal column action potentials. SEPs are simultaneously elicited via stimulation of distal peripheral nerves and are captured using the standard SEP scalp recording montage. Stimulation through select paddle contacts that overlie the same dorsal column that is activated by the peripheral stimulus, and that is also coincident with the ascending SEPs, will result in attenuation of the cortically recorded response. This is due to the “collision” of the descending antidromic potentials elicited proximally in the spinal cord with the ascending orthodromic SEP elicited distally in the periphery. This technique helps in the selection of the optimal paddle contacts for treatment [324–326]**.**

Surgical examples include the following: mapping for removal of intermedullary tumors [40, 244, 319, 320] and vascular malformations [244]**,** and placement of spinal neuromodulation devices [324–326]**.**

#### Brainstem and thalamic mapping

SEP pathways traverse the brainstem as they project up to the thalamus. Occasionally, surgery in and around the brainstem risks damage to these pathways and the acquisition of SEPs are a useful monitoring modality. However, in most cases, monitoring of SEPs is complementary to and perhaps of secondary importance to the monitoring of the function of various cranial nerves. SEPs have been successfully used for functional mapping of cavernous malformations [326]. SEPs can also be used to determine a safe location for making a thalamic lesion or implanting a deep brain stimulator in the thalamus for alleviating tremor in patients with Parkinson’s disease.

Surgical examples include the following: craniotomy for removal of CP angle tumor [318, 327–333], fourth ventricle lesions [334], thalamotomy for decrease of Parkinsonian tremor [44, 335].

#### Cortical mapping

When a brain lesion is located near the sensory-motor areas, it places these eloquent tissues at surgical risk. When removing a tumor, the surgical objective is to remove as much tumor as possible and to spare primary neural function, often prioritizing the motor area. It can be difficult to identify and/or delineate these eloquent areas based on visual inspection of the cortical surface alone. Neurophysiological mapping using SEPs provides a functional guide to the anatomy. Because of their typically large amplitude and reproducible waveforms, recordings of upper extremity SEP are widely used for this purpose. They demonstrate polarity inversion across the central sulcus, known as phase reversal. The sensory responses are recorded above the sensory and motor cortices.

SEPs from lower extremity nerves may also demonstrate phase reversal, although not as reliably as those from the upper extremity [336]. Lower extremity phase reversal, though rare, is most commonly observed within the mesial, interhemispheric cortex [337–341].

SEP responses are recorded directly from the brain surface using a strip or grid of recording electrodes. By recording the SEP responses from each electrode contact, the site(s) where polarity inversion occurs is determined, indicating the location of the underlying sensory and possible motor areas [111, 342–344]. If a phase reversal is absent or indiscernible, the SEP map is still helpful in localizing the functional cortex. For example, the contact exhibiting the maximum amplitude response is presumed nearest to the central sulcus [48, 49]. When lower extremity nerves are used for sensory cortical mapping, the maximum amplitude criteria is often the only usable parameter [341]. Finally, gaining additional details regarding the waveforms can aid in the precise characterization of the cortical homunculus. For example, capturing a triphasic response for the median and ulnar nerves indicates the electrode is positioned on the brain surface directly over the neurophysiological generators for those nerves; thus, permitting the capture of the additional P25 peak [79, 345–347].

Adding a scalp derivation, such as those typically used for SEP monitoring, to the cortical mapping montage helps to discern the polarity of the cortically recorded responses. It is important to remember that, due to the paradoxical laterization observed for posterior tibial SEPs, P37 is observed ipsilateral to the activated limb because the dipole is towards that side. When performing lower SEP sensory mapping, one should take into consideration paradoxical lateralization [341, 348–351].

Activating the SEP pathway for cortical mapping purposes is the same as for IONM monitoring. The appropriate nerve depends on the location of the surgery and the tissue at risk. The stimulated limb is contralateral to the side of the surgery. An upper extremity versus a lower extremity nerve may be selected roughly for lateral versus medial mapping. Due to the high signal-to-noise ratio, the required number of trials per average for mapping is much less than for monitoring. For recording, typically, each contact of the electrode serves as the active input of the derivation, and a scalp electrode, such as Fpz or Fz, serves as the reference. Less commonly, each electrode contact is paired with the adjacent contact in a bipolar montage. The referential montage is preferred for the more stereotypical responses that make the determination of a phase reversal more apparent. However, the bipolar montage provides more focal information. The filters and amplifier settings should be adjusted accordingly. The vertical display parameters should be adjusted, taking into consideration the larger amplitude responses that are commonly recorded from the brain surface compared to scalp-recorded SEPs [79].

Sensory mapping has and continues to be widely utilized to identify the central sulcus, and as a result, its use has helped to efficiently localize the primary motor cortex as well. In some centers, where pre-operative functional imaging and cortical mapping are extensively utilized, the use of sensory mapping is largely being supplanted by the sole use of cortical stimulation for mapping of the motor cortex directly. However, when clear motor responses cannot be obtained, the use of sensory mapping becomes indispensable for localization of eloquent tissues [352].

Surgical examples include the following: mapping of the sensory, motor and/or language cortices for tumor/cystic lesion removal [48, 79, 111, 336, 337, 341–343, 352], for repair of arterial and venous malformations [79, 336, 337], placement of aneurysm clips [337], and resection of epileptogenic foci [348, 353].

## Interpretation and correlation with outcomes

### Alarm or alert criteria

Despite the use of SEP monitoring for over four decades, the designation of appropriate alarm or warning criteria for their use remains controversial. Early reports regarding the use of SEPs for spinal cord monitoring suggested a 10% increase in latency of the primary SEP cortical response (i.e., N20 or P37), and/or a decrease of more than 50% in cortical peak to peak amplitude from baseline that is sustained for more than 10 min should be considered alarm criteria for the possible onset of a neurologic compromise and a basis for intervention [3, 354–356]. Over time, these alarm criteria became the traditional alarm or warning criteria for SEP changes. However, they were largely established based on empirical findings and, as they came to be more widely utilized, there were reports that some patients can routinely have EP changes which exceed these alarm criteria without any postoperative deficits [297, 357–359]. Since that time, it has been suggested that the traditional alarm criteria overemphasize latency prolongation and fail to consider baseline drift or reproducibility [22, 50]. If baseline drift is not taken into account, false positives or negatives can arise when amplitude decrements are compared to early baseline responses rather than more recent pre-change response amplitudes [22, 50].

The magnitude of an amplitude decrement needed to be significant and clearly non-random varies with established reproducibility. The reproducibility of SEPs can markedly influence the reliability of monitoring. A 50% amplitude loss has been considered an appropriate warning criterion but, in some cases, its use risks false negative findings with surgically related decrements less than 50% or false positives with non-reproducible signals [50]. Thus, some have argued that when reproducibility is high, warning criteria of less than 50% can be effectively used [50]. Recently, recommended adaptive warning criteria have included visually obvious amplitude reductions from recent pre-changed values and clearly exceeding variability, particularly when abrupt and focal [22]. It has also been suggested that warning criteria should be different for healthy patients and those with impaired spinal cord function [215] because patients with preexisting neurological deficits tend to have unstable and variable SEPs in terms of their latency and amplitude measurements [215, 355, 359]. In some patients, amplitude variability is greater than 50% and such spontaneous variations in SEP amplitude are sufficient to obscure those caused by surgical intervention [359]. In addition, there may be certain patients or situations for which reliable monitoring cannot be accomplished and the use of the simple warning criteria for significant intraoperative SEP changes (10% latency increase, 50% amplitude loss) is relatively ineffective [359]. For such patients with high variability and weak amplitudes, it is suggested that they may not be well protected by SEP monitoring [359]. Such may be the case even when recording montages have been optimized with regard to signal-to-noise ratios. Nevertheless, since early on, the traditional arbitrarily set warning criteria for SEP response changes continue to be referred to and utilized even to the present day [356, 360–362]. Despite their empirical basis as alert criteria, when such changes do occur, they should be considered a cause for concern resulting in heightened vigilance.

The risk of a clinical deficit associated with a pathologic decrement varies with its reversibility. Quickly reversible (less than 30 to 40 min) decrements usually, but do not always, predict the absence of new postoperative deficits. However, such deficits become more likely with protracted (greater than 40 to 60 min) and especially irreversible decrements [22, 191, 359, 363].

### Confounding factors

It has long been recognized that spontaneous variations in SEP waveforms occur, and these variations may complicate SEP interpretation and do not necessarily imply surgical neurologic system trespass [364]. A variety of systemic and local factors can cause SEP variation, and the impact of some primary causative factors—anesthetic agents and physiological parameters—is discussed above (see Sect. 6). A local factor well-recognized to impact SEP waveforms is regional temperature changes, such as from cold irrigation-fluid [195]. In addition, SEP response variability has been found to be a function of patient diagnosis, neuromuscular status, age, and procedural approach during spine surgery [359]. If the degree of variability is large, it may in some cases severely limit the reliability and usefulness of spinal cord monitoring in detecting early cord compromise [359]. Confounding factors and their contribution to spontaneous variations in SEP waveforms emphasizes the necessity of considering the full context of intraoperative factors, medical comorbidities, and surgical events in the interpretation of SEPs.

### Clinical outcome analysis

The ideational framework for interpreting SEP results can be equivocal, particularly when considering reversible signal changes [365]. Conceptually, true positive results and true negative SEP results are straightforward. A true positive describes a situation in which there is a persistent significant change in the evoked potential and, upon anesthetic emergence, the patient exhibits a postoperative neurologic deficit in the corresponding anatomic area. For a true negative, on the other hand, there are no significant changes in the evoked potential during the case and no corresponding postoperative deficits. Confusion has ensued in the literature over false positives and false negatives, however [209, 219]. A significant change in evoked potential that leads to a change in surgical and/or physiological management (e.g., elevation of the blood pressure) with a resulting improvement in the evoked potential and no postoperative deficit is a true positive (or could be called a transient true positive or reversible true positive) since the report of the signal change assisted in patient management to avert permanent postoperative injury. This is not a false positive because a unique, customized patient management strategy was triggered by the IONM alert, that then demonstrated reversibility and no correlative postoperative neurologic deficit [209]. Biological plausibility, temporal association, and strength of association can all be used to support causation between management, intervention and evoked potential recovery/reversibility [365]. Another example of a transient true or reversible true positive exists in the case of emerging peripheral nerve injuries related to positioning [265]. A signal change that triggers an adjustment of patient positioning with improvement or resolution of the evoked potential and no postoperative peripheral nerve dysfunction would be a true positive. Finally, a postoperative neurological deficit in a pathway not monitored by SEPs is not a false negative (the modality simply cannot assess for that deficit, such as in an isolated motor pathway). In this instance, the need for multimodality and multi-foci monitoring to increase IONM sensitivity is thus highlighted [219].

## Safety and technical considerations

### Electrical safety and maintenance

The selection and operation of any device used for neuromonitoring purposes should conform to the recommendations set forth by the ASNM [366], AEEGS [13], ASET [14], and the ACNS [367]. Interested parties are encouraged to review the appropriate sections contained in these documents and other publications [100]. Routine equipment maintenance, the evaluation of leakage current, and an inspection of the overall electrical integrity of the equipment should be routinely performed on a regular basis, as required by the manufacturer or by the biomedical engineering protocol at a given institution [100]. In cases when faulty or malfunctioning equipment is suspected, the equipment should not be used until an inspection and any necessary repair has been performed.

### General infection control guidelines

General infection control procedures for personnel, equipment and electrodes should be consistent with those previously published [100, 366] as well as the policy and procedures of the individual institution. Equipment used in the OR should be protected from contamination or exposure to body fluids. Neuromonitoring and ancillary equipment such as cables and the boxes used for stimulation and recording purposes should be cleaned with an appropriate disinfectant after each case. Disposable subdermal needle electrodes once used should be disposed of in the appropriate sharps’ disposal container.

### Risks

The use of subdermal needle electrodes for both stimulation and recording has become commonplace largely because of their effectiveness and OR time constraints. However, as discussed earlier, their use is associated with risks which include needle stick injuries with possible infections for the monitoring and other hospital staff members and/or the patient [14, 50, 368, 369]. IONM personnel should adhere to standard precautions which guard against the risk of accidental exposure to blood and bodily fluids.

For the patient, burns can occur at the electrode sites if the electrocautery device is not properly grounded [94, 369, 370]. In addition, invasive subdural or epidural electrodes often used for spinal recordings and brain or dorsal column mapping, may be associated with risks of hemorrhage, trauma, or infection [50].

## Documentation

### Chart note

A report or chart note should be generated for the patient’s medical record indicating that monitoring was performed during the surgical procedure. The report or chart note should describe what function was monitored, how the monitoring was performed, what information the monitoring provided, and should also include any other information that was relevant to the medical status of the patient. Any information relevant to the well-being of the patient must be shared with other health care professionals for continuing care reasons. Therefore, the report should be completed as soon as possible. Even if this report is not completed prior to the patient leaving the OR, the neuromonitoring team should be certain that all relevant monitoring data has been communicated to the physicians caring for the patient. Specifically, aside from information conveyed during the surgical procedure, this should include the status of the monitored responses relative to the baseline responses obtained during the surgical procedure.

### Monitoring data

All the SEP data traces and other information that are acquired during monitoring should be saved electronically and/or printed for possible later review. The monitoring records should include detailed information such as demographic data, diagnosis and type of surgery, equipment and neuromonitoring procedures, neuromonitoring personnel, intraoperative events, and clinical outcome, if available. When possible, great care should be taken to acquire artifact free SEP responses prior to, during, and after various routine and critical surgical events. In addition, relevant physiological variables (e.g. blood pressure, temperature), anesthetic agents and levels, significant SEP changes, any critical alerts or alarms to the surgeon and anesthesia provider, the event log (electronic comments entered by the technologist) and the chat log (real-time conversation between the technologist and individual providing professional IONM oversight), the responses of the surgeon to any data supplied and any interventions or changes in surgical or anesthetic care based on the IONM should all be appropriately documented in the electronic file or on the hardcopy, if needed, of the SEP response traces and/or the log of the neuromonitoring remarks for each patient [12, 13, 366, 371]. Though the requirements of what data needs to be saved, where it is to be saved, and for how long is dictated by state law, and that some hospital’s policies regarding medical record storage exceed state requirements, it is recommended that all data be saved. The Centers for Medicare and Medicaid Services (CMS) determine the medical record retention policy at the federal level. It requires medical facilities to maintain medical records for seven years from the date of service. The facility, itself, doesn’t have to do the record-keeping as it can be done by a third party [372]**.**

### Structure of the neuromonitoring team

#### Staffing practice patterns

Staffing models for IONM vary greatly across institutions. IONM may be divided into two levels of service delivery: professional/supervisory and technical [209]. The ASNM recognizes the importance of appropriately qualified IONM personnel to provide professional oversight as well as to perform the monitoring tasks. Individuals performing or supervising IONM services should have gained appropriate education, training, experience, and certification of competency prior to practicing in a clinical setting. The ASNM has published IONM personnel qualifications. In order to address this issue, the ASNM has published practice guidelines for the supervising professionals overseeing IONM and refers the reader to these documents [366, 373–377].

#### Credentials of the neuromonitoring team

A recently published joint position statement by the ASNM, ACNS, American Association of Neuromuscular and Electroneurodiagnostic Medicine (AANEM) and ASET provides the guidelines for qualifications of neurodiagnostic personnel [377].

As referenced in these guidelines, there are several organizations which offer credentials at the professional/supervisory level. These include the American Board of Neurophysiologic Monitoring (ABNM), which grants recognition as a Diplomate (DABNM), the American Board of Psychiatry and Neurology (ABPN) which grants a status as “Certification in the Subspecialty of Clinical Neurophysiology”, the American Board of Clinical Neurophysiology (ABCN) which grants a certification “with special competency in intraoperative neuromonitoring”, and the American Board of Electrodiagnostic Medicine (ABEM) which provides a Diplomate certification in neurophysiology concentrating on EMG and evoked potentials. The American Speech-Language-Hearing Association’s (ASHA)’s American Audiology Board of Intraoperative Monitoring (AABIOM) offers BCS-IOM (Board Certification in Intraoperative Monitoring) [378].

Also referenced in the joint guidelines [377] for those seeking certification at the technical level, the American Board of Registry for Electroneurodiagnostic Technologists (ABRET) offers Certification in Intraoperative Monitoring (CNIM). Criteria for ABRET certification can be found at their website [379]. ASET has also published national competency skill standards for performing IONM [376].

In addition to having appropriate credentials and demonstrating competency in IONM, the ASNM recognizes the value of continuing education, as well as the development of institutional policies and procedures which include scope-of-practice, duties related to both technical and professional aspects of practice, and interpersonal communications.

## Summary and recommendations

This ASNM position statement can be interpreted as providing guidelines for the acquisition and application of intraoperative SEP responses. Guidelines are recommendations for patient management that may identify a particular strategy or range of management strategies that reflect moderate clinical certainty [366]. The recommendations of the ASNM regarding the use of SEPs are based on a standardized set of terminology adopted for evaluating the strength of evidence and the grades of recommendations [22, 380].

The definitions of the quality of evidence ratings and the strength of recommendation ratings are as follows:

- Class I. Evidence provided by one or more well-designed, prospective, blinded, controlled clinical studies.
- Class II. Evidence provided by one or more well-designed clinical studies such as case control, cohort studies, etc.
- Class III. Evidence provided by expert opinion, non-randomized historical controls, or case reports of one or more.

The strength of recommendation ratings are as follows:

- Type A. Strong positive recommendation, based on Class I evidence, or overwhelming Class II evidence.
- Type B. Positive recommendation, based on Class II evidence.
- Type C. Positive recommendation, based on strong consensus of Class III evidence.
- Type D. Negative recommendation, based on inconclusive or conflicting Class II evidence.
- Type E. Negative recommendation, based on evidence of ineffectiveness or lack of efficacy.
- Type U: No recommendation, based on divided expert opinion or insufficient data.
  A. The acquisition and interpretation of intraoperative SEPs should be performed by individuals (Class III evidence, strong Type C recommendation) with the technical and professional qualifications specified in the ASNM guidelines published in 2019 and the more recent joint guidelines published by the ASNM, ACNS, AANEM, and ASET [375, 377]**.**
  B. Based on current clinical literature and clinical and scientific evidence, SEPs are an established intraoperative monitoring modality for either localizing the human sensorimotor cortex and dorsal columns or assessing the function of the somatosensory pathways during surgical procedures in the spinal cord and brain. (Class II and III evidence, Type A recommendation)
  C. On the basis of current clinical literature and the opinions of most experts, SEPs have limitations as an intraoperative monitoring tool. These include the following:
    1. SEPs are an effective means of monitoring cortical function during various cerebrovascular surgical procedures (i.e., carotid endarterectomies, clipping of intracranial aneurysms of the anterior vessels of the circle of Willis). Other monitoring techniques such as analog and computer-processed EEG, MEPs and/or transcranial doppler techniques may provide additional information in the appropriate clinical situation (Class II and III evidence, Type B recommendation)
    2. SEPs may provide indirect information about motor pathway function. Other techniques that directly monitor motor pathway function may provide additional information in the appropriate clinical situation. (Class II and III evidence, Type B recommendation)
    3. SEPs are affected by commonly used anesthetic drugs and physiological parameters. This is particularly true for cortical SEP responses and less so for subcortical and peripheral responses. Monitoring of spinal cord and cerebral function should include the following:
      - The use of cortical and subcortical recording sites. (Class II evidence, Strong Type B recommendation)
      - Documentation of anesthetic dosages and physiological parameters. (Class II evidence, Strong Type B recommendation)
    4. The sensitivities of mixed nerve SEPs and dermatomal SEPs (DSEPs) for assessing spinal nerve root function are controversial (Class III evidence, Type E recommendation). Other techniques may be more efficacious in monitoring nerve root function in the appropriate clinical situation.

## Data Availability

No datasets were generated or analysed during the current study.
